# Characterising social contacts under COVID-19 control measures in Africa

**DOI:** 10.1186/s12916-022-02543-6

**Published:** 2022-10-12

**Authors:** Zlatina Dobreva, Amy Gimma, Hana Rohan, Benjamin Djoudalbaye, Akhona Tshangela, Christopher I. Jarvis, Kevin van Zandvoort, Matthew Quaife

**Affiliations:** 1 Faculty of Public Health and Policy, London School of Hygiene and Tropical Medicine, London, UK; 2 Faculty of Epidemiology and Population Health, London School of Hygiene and Tropical Medicine, London, UK; 3 UK Public Health Rapid Support Team, London School of Hygiene and Tropical Medicine, London, UK; 4 Africa Centres for Disease Control and Prevention, Addis Ababa, Ethiopia

**Keywords:** COVID-19, SARS-CoV-2, Social contacts, Physical distancing, Modelling

## Abstract

**Background:**

Early in the COVID-19 pandemic, countries adopted non-pharmaceutical interventions (NPIs) such as lockdowns to limit SARS-CoV-2 transmission. Social contact studies help measure the effectiveness of NPIs and estimate parameters for modelling SARS-CoV-2 transmission. However, few contact studies have been conducted in Africa.

**Methods:**

We analysed nationally representative cross-sectional survey data from 19 African Union Member States, collected by the Partnership for Evidence-based Responses to COVID-19 (PERC) via telephone interviews at two time points (August 2020 and February 2021). Adult respondents reported contacts made in the previous day by age group, demographic characteristics, and their attitudes towards COVID-19. We described mean and median contacts across these characteristics and related contacts to Google Mobility reports and the Oxford Government Response Stringency Index for each country at the two time points.

**Results:**

Mean reported contacts varied across countries with the lowest reported in Ethiopia (9, SD=16, median = 4, IQR = 8) in August 2020 and the highest in Sudan (50, SD=53, median = 33, IQR = 40) in February 2021. Contacts of people aged 18–55 represented 50% of total contacts, with most contacts in household and work or study settings for both surveys. Mean contacts increased for Ethiopia, Ghana, Liberia, Nigeria, Sudan, and Uganda and decreased for Cameroon, the Democratic Republic of Congo (DRC), and Tunisia between the two time points. Men had more contacts than women and contacts were consistent across urban or rural settings (except in Cameroon and Kenya, where urban respondents had more contacts than rural ones, and in Senegal and Zambia, where the opposite was the case). There were no strong and consistent variations in the number of mean or median contacts by education level, self-reported health, perceived self-reported risk of infection, vaccine acceptance, mask ownership, and perceived risk of COVID-19 to health. Mean contacts were correlated with Google mobility (coefficient 0.57, *p*=0.051 and coefficient 0.28, *p*=0.291 in August 2020 and February 2021, respectively) and Stringency Index (coefficient −0.12, *p* = 0.304 and coefficient −0.33, *p*=0.005 in August 2020 and February 2021, respectively).

**Conclusions:**

These are the first COVID-19 social contact data collected for 16 of the 19 countries surveyed. We find a high reported number of daily contacts in all countries and substantial variations in mean contacts across countries and by gender. Increased stringency and decreased mobility were associated with a reduction in the number of contacts. These data may be useful to understand transmission patterns, model infection transmission, and for pandemic planning.

**Supplementary Information:**

The online version contains supplementary material available at 10.1186/s12916-022-02543-6.

## Background

Over 352 million cases and 5.6 million deaths from coronavirus disease (COVID-19) have been recorded worldwide as of January 24, 2022 [1]. With the first case on the African continent reported in Egypt on February 14, 2020 [2], Africa accounts for 3.4 and 4.2% of measured COVID-19 cases and deaths, respectively [3]. While some countries have reported more infections than others, with South Africa, Morocco, Tunisia, Ethiopia, Egypt, Libya, Kenya and Zambia accounting for nearly 70% of all COVID-19 cases in Africa by January 24, 2022 [3], the relatively low burden of COVID-19 in Africa compared to Europe and North America has been, in part, attributed to demographic factors such as younger and more rurally located populations, differences in case and death detection capacity, and environmental factors such as higher temperatures [4–6], as well as countries’ previous experience with outbreak prone diseases [7].

In the absence of effective pharmaceutical interventions early in the pandemic, non-pharmaceutical interventions (NPIs), or public health and social measures, were adopted to limit transmission and person-to-person contacts. African countries’ success in containing and delaying the first wave of SARS-CoV-2 infections was partially attributed to the prompt and early introduction of NPIs. However, the timing and intensity of those measures varied between countries. By March 16, 2020, when the total reported cases had reached 64, South Africa declared a national disaster, banned travel from the worst-affected countries and public gatherings, and closed schools. By March 26, 2020, when cases neared 927 and deaths were zero, the government announced a three-week national lockdown [8]. Closure of educational institutions and travel restrictions were also implemented in Kenya, followed by a partial lockdown on April 6, 2020, with 158 cases and 6 deaths [8]. In the second half of March 2020, a 48-h lockdown was also announced in DRC’s second largest city following the identification of two cases, whereas Senegal imposed a dusk-to-dawn curfew from March 24, 2020, with 79 cases and zero reported deaths [9]. In contrast, Ethiopia did not introduce lockdown measures but relied on an extensive testing and screening programme combined with public awareness campaigns [10].

Given the current challenge of ensuring sustained access to vaccines, many African countries continue to rely on NPIs, which can be economically burdensome, unsustainable, or challenging for people to adhere to. The effectiveness of many control measures can be assessed through social contact studies, which estimate the number and type of person-to-person contacts. These contact estimates are also important components of mathematical models of respiratory infectious disease dynamics [11]. Few contact studies have been conducted in Africa, which means that models must rely on estimates from so-called synthetic matrices, which adjust high-income country contact patterns with population and household structures in low- and middle-income countries (LMICs) [12]. Exceptions to this since the start of the pandemic are a study in informal settlements in Kenya [13], one in KwaZulu-Natal in South Africa [14], and one in different settings in Ethiopia [15]. However, these are not nationally representative. This is also the case for other pre-COVID-19 contact studies—in rural coastal Kilifi in Kenya [16, 17], rural Uganda [18], rural Senegal [19], in Cape Town and in a rural community and urban area in South Africa [20, 21], and in a rural and a peri-urban site of Manicaland in Zimbabwe [22].

We used cross-sectional data collected by the Partnership for Evidence-based Responses to COVID-19 (PERC) and described and quantified social contacts from nationally representative samples of Africa Union Member States gathered from August 4 to 17, 2020 and February 8 to 25, 2021. We compared mean and median contacts by different demographic characteristics and COVID-19 perceptions. We related contact patterns to the intensity of restrictions during the survey periods using the Oxford Government Response Stringency Index as well as Google Community Mobility Reports. We further considered how well these indicators capture changes in contacts. This paper provides one of the first comprehensive measurements of person-to-person contact behaviour across Africa during the COVID-19 pandemic.

## Methods

### Survey methodology

Telephone interviews were conducted with adult respondents (aged 18 and over) with access to a landline or a mobile phone in 18 countries (Cameroon, Democratic Republic of Congo, Egypt, Ethiopia, Ghana, Guinea, Ivory Coast, Kenya, Liberia, Mozambique, Nigeria, Senegal, South Africa, Sudan, Tunisia, Uganda, Zambia, and Zimbabwe) during August 4 to 17, 2020 (survey 1, S1) and these countries plus Morocco during February 2 to 28, 2021 (survey 2, S2). The survey was designed as a quota sample survey through Random Digit Dial (RDD) with quotas on gender and urban/rural location within each country to achieve a sample closely matching the profile of each country. Multiple call-backs to unanswered numbers dialled was not part of the design [23].

Respondents were first asked questions relating to their knowledge about COVID-19, their risk perceptions of catching COVID-19, their general health, and the potential impact of SARS-CoV-2 on their heath (Additional file 1). They were subsequently asked about their attitude towards the existing control measures, their confidence in the government’s response, their attitude towards vaccination (in survey 2 only) and the burden of the pandemic on them and their household. Respondents were finally asked whether they had been diagnosed or believed to have had COVID-19, about their socio-demographic characteristics, and the number of contacts they had had in the last 24 h with contactees (people who respondents had a contact with) aged 0–17, 18–55, or over 55. A contact was defined as “anyone with whom [the respondent] exchanged at least a few words and was close enough to not need to raise [their] voice or [who they] had direct physical contact with (including handshaking or other contact).” Contacts were categorised according to contacts with members of the household; with external visitors to the household; with people at work, school, or university; or with people in other places. Demographic data such as the respondent’s self-reported age, residence, household size, monthly income, and educational level were also collected.

### Statistical analysis

R version 4.1.1 and Stata version 16.1 were used for the cleaning and preparation of the survey datasets and the subsequent analysis [24, 25]. Data analysis was performed at the level of individual countries due to the anticipated variability between countries and given the novelty of such country-level analysis.

Respondents’ gender, age, residence area, household head education level, monthly income and household size were characterised (Table 1 and Additional file 2: Table S1 for surveys 1 and 2, respectively). Survey 2 had not collected monthly income in a common currency, preventing comparisons across countries. The age structure of the sampled populations was also compared to United Nations’ World Population Prospects (WPP) data on the number of people by age group (Additional file 2: Table S2) and the percentage of female and rural population according to World Bank estimates (Additional file 2: Table S3) in each country to assess the representativeness of the sample [26].

**Table 1:** Survey 1 respondent descriptives

	Cameroon *n*=1449	DRC *n*=1351	Egypt *n*=1206	Ethiopia *n*=1571	Ghana *n*=1338	Guinea *n*=1283	Ivory Coast *n*=1416	Kenya *n*=1123	Liberia *n*=1366	Mozambique *n*=1314	Nigeria *n*=1304	Senegal *n*=1259	South Africa *n*=1395	Sudan *n*=1438	Tunisia *n*=1218	Uganda *n*=1286	Zambia *n*=1290	Zimbabwe *n*=1333
**Gender** ***n*** **(col %)**																		
Female	698 (48%)	637 (47%)	603 (50%)	623 (40%)	675 (50%)	538 (42%)	640 (45%)	570 (51%)	662 (48%)	568 (43%)	644 (49%)	626 (50%)	714 (51%)	750 (52%)	611 (50%)	638 (50%)	629 (49%)	658 (49%)
Male	751 (52%)	714 (53%)	603 (50%)	948 (60%)	663 (50%)	745 (58%)	776 (55%)	553 (49%)	704 (52%)	746 (57%)	660 (51%)	633 (50%)	681 (49%)	688 (48%)	607 (50%)	648 (50%)	661 (51%)	675 (51%)
**Age group** ***n*** **(col %)**																		
19–29	789 (54%)	610 (45%)	592 (49%)	791 (50%)	797 (60%)	804 (63%)	493 (35%)	493 (44%)	655 (48%)	765 (58%)	550 (42%)	535 (42%)	463 (33%)	928 (65%)	248 (20%)	488 (38%)	862 (67%)	446 (33%)
30–39	425 (29%)	429 (32%)	315 (26%)	477 (30%)	392 (29%)	290 (23%)	573 (40%)	364 (32%)	408 (30%)	348 (26%)	520 (40%)	427 (34%)	473 (34%)	298 (21%)	281 (23%)	382 (30%)	213 (17%)	387 (29%)
40–49	158 (11%)	202 (15%)	170 (14%)	183 (12%)	96 (7%)	98 (8%)	239 (17%)	163 (15%)	186 (14%)	132 (10%)	191 (15%)	185 (15%)	271 (19%)	137 (10%)	295 (24%)	204 (16%)	129 (10%)	274 (21%)
50–59	53 (4%)	81 (6%)	102 (8%)	71 (5%)	35 (3%)	59 (5%)	79 (6%)	80 (7%)	78 (6%)	44 (3%)	35 (3%)	73 (6%)	129 (9%)	55 (4%)	208 (17%)	124 (10%)	76 (6%)	140 (11%)
60	24 (2%)	29 (2%)	27 (2%)	49 (3%)	18 (1%)	32 (2%)	32 (2%)	23 (2%)	39 (3%)	25 (2%)	8 (1%)	39 (3%)	59 (4%)	20 (1%)	186 (15%)	88 (7%)	10 (1%)	86 (6%)
Area																		
Rural	809 (56%)	626 (46%)	658 (55%)	956 (61%)	675 (50%)	641 (50%)	597 (42%)	857 (76%)	653 (48%)	778 (59%)	722 (55%)	697 (55%)	589 (42%)	752 (52%)	397 (33%)	977 (76%)	758 (59%)	870 (65%)
Urban	640 (44%)	725 (54%)	548 (45%)	615 (39%)	663 (50%)	642 (50%)	819 (58%)	266 (24%)	713 (52%)	536 (41%)	582 (45%)	562 (45%)	806 (58%)	686 (48%)	821 (67%)	309 (24%)	532 (41%)	463 (35%)
**Household head education n (col %)**																		
No education	56 (4%)	10 (1%)	74 (6%)	326 (21%)	104 (8%)	386 (30%)	329 (23%)	77 (7%)	179 (13%)	146 (11%)	21 (2%)	351 (28%)	38 (3%)	119 (8%)	248 (20%)	311 (24%)	38 (3%)	49 (4%)
Primary	430 (30%)	71 (5%)	105 (9%)	299 (19%)	224 (17%)	224 (17%)	470 (33%)	204 (18%)	134 (10%)	382 (29%)	85 (7%)	279 (22%)	273 (20%)	244 (17%)	489 (40%)	514 (40%)	145 (11%)	212 (16%)
Secondary	298 (21%)	326 (24%)	352 (29%)	208 (13%)	394 (29%)	63 (5%)	161 (11%)	346 (31%)	359 (26%)	420 (32%)	522 (40%)	189 (15%)	693 (50%)	310 (22%)	147 (12%)	144 (11%)	323 (25%)	551 (41%)
Tertiary	326 (22%)	634 (47%)	518 (43%)	596 (38%)	530 (40%)	377 (29%)	212 (15%)	439 (39%)	606 (44%)	308 (23%)	613 (47%)	276 (22%)	337 (24%)	648 (45%)	221 (18%)	285 (22%)	701 (54%)	389 (29%)
Post-graduate	261 (18%)	292 (22%)	134 (11%)	106 (7%)	50 (4%)	204 (16%)	219 (15%)	37 (3%)	82 (6%)	23 (2%)	58 (4%)	114 (9%)	27 (2%)	105 (7%)	102 (8%)	17 (1%)	69 (5%)	83 (6%)
Missing	78 (5.4%)	18 (1.3%)	23 (1.9%)	36 (2.3%)	36 (2.7%)	29 (2.3%)	25 (1.8%)	20 (1.8%)	6 (0.4%)	35 (2.7%)	5 (0.4%)	50 (4.0%)	27 (1.9%)	12 (0.8%)	11 (0.9%)	15 (1.2%)	14 (1.1%)	49 (3.7%)
**Monthly income (USD) n (col %)**																		
$0–$100	355 (24%)	329 (24%)	153 (13%)	639 (41%)	287 (21%)	496 (39%)	282 (20%)	509 (45%)	804 (59%)	497 (38%)	343 (26%)	126 (10%)	166 (12%)	235 (16%)	106 (9%)	812 (63%)	435 (34%)	1109 (83%)
$101–$500	583 (40%)	557 (41%)	750 (62%)	666 (42%)	642 (48%)	449 (35%)	808 (57%)	389 (35%)	395 (29%)	359 (27%)	576 (44%)	438 (35%)	389 (28%)	793 (55%)	698 (57%)	318 (25%)	647 (50%)	84 (6%)
$501–$2000	116 (8%)	193 (14%)	90 (7%)	95 (6%)	149 (11%)	71 (6%)	197 (14%)	74 (7%)	43 (3%)	31 (2%)	74 (6%)	213 (17%)	254 (18%)	235 (16%)	302 (25%)	40 (3%)	125 (10%)	13 (1%)
Over $2000	10 (1%)	85 (6%)	6 (0%)	16 (1%)	13 (1%)	12 (1%)	8 (1%)	8 (1%)	16 (1%)	5 (0%)	31 (2%)	70 (6%)	44 (3%)	18 (1%)	19 (2%)	4 (0%)	5 (0%)	8 (1%)
Missing	385 (26.6%)	187 (13.8%)	207 (17.2%)	155 (9.9%)	247 (18.5%)	255 (19.9%)	121 (8.5%)	143 (12.7%)	108 (7.9%)	422 (32.1%)	280 (21.5%)	412 (32.7%)	542 (38.9%)	157 (10.9%)	93 (7.6%)	112 (8.7%)	78 (6.0%)	119 (8.9%)
**Age**																		
Median [IQR]	28 [12]	30 [13]	30 [17]	29 [11]	28 [8.0]	26 [12]	33 [12]	31 [13]	30 [13]	28 [12]	30 [11]	31 [13]	35 [16]	26 [11]	41 [22]	32 [17]	26 [11]	34 [18]
**Household size**																		
Median [IQR]	5.0 [4.0]	6.0 [4.0]	5.0 [2.0]	5.0 [3.0]	5.0 [4.0]	7.0 [5.0]	6.0 [4.0]	5.0 [3.0]	7.0 [4.0]	5.0 [3.0]	6.0 [3.0]	9.0 [6.0]	5.0 [3.0]	7.0 [4.0]	4.0 [2.0]	6.0 [4.0]	6.0 [3.0]	5.0 [2.0]

Legend: The table shows the number (*n*) and percentage of total participants (col%) in a given country by gender, age group, household head education level, monthly income in US dollars, as well as the median and interquartile range (IQR) of the participants’ age and the reported size of the household they live in

**Fig. 1:**
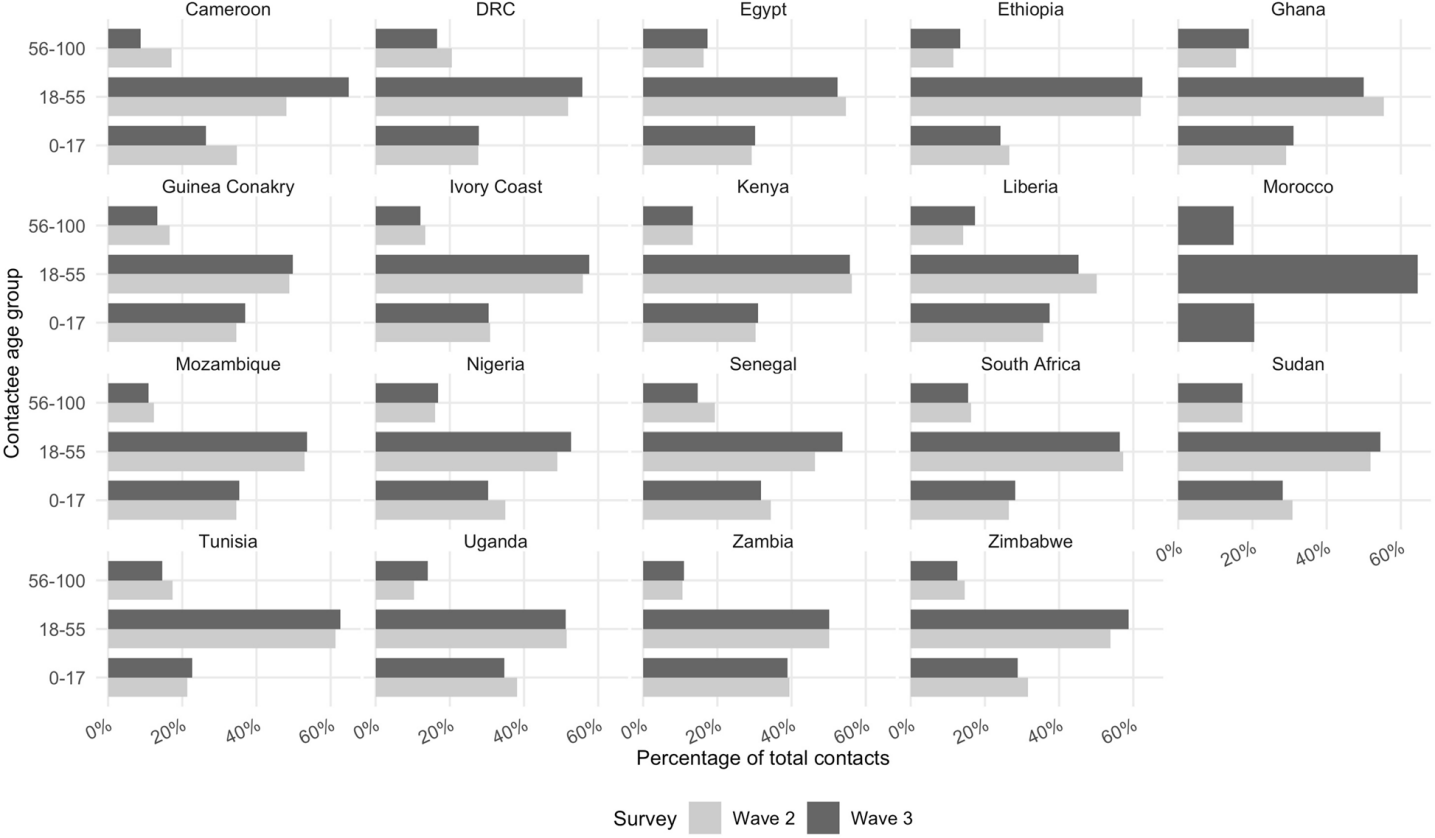
Percentage of contacts out of total average recorded contacts by contactees' age group and country in surveys 1 and 2

**Fig. 2:**
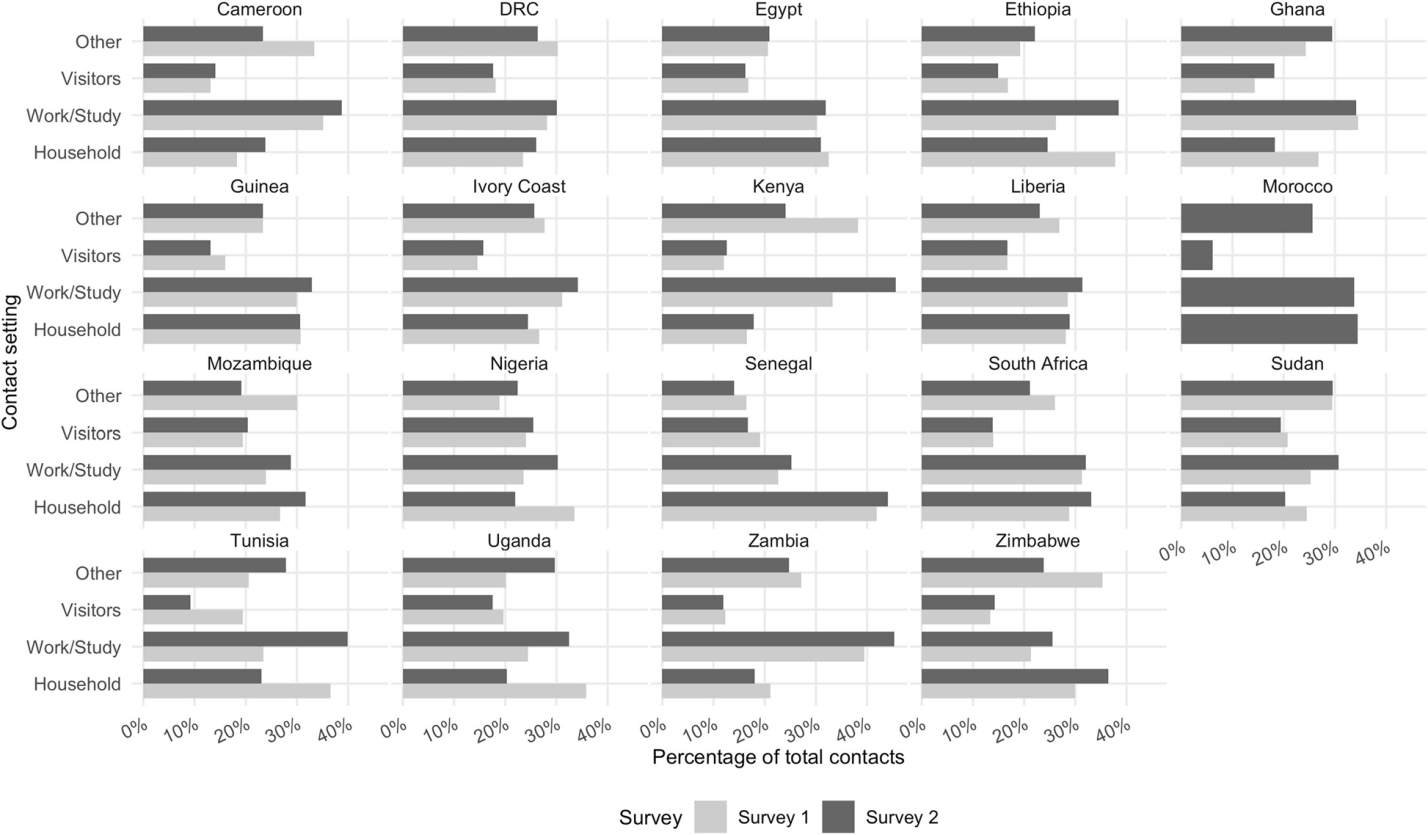
Percentage of contacts out of total average recorded contacts by contact setting and country in surveys 1 and 2

**Fig. 3:**
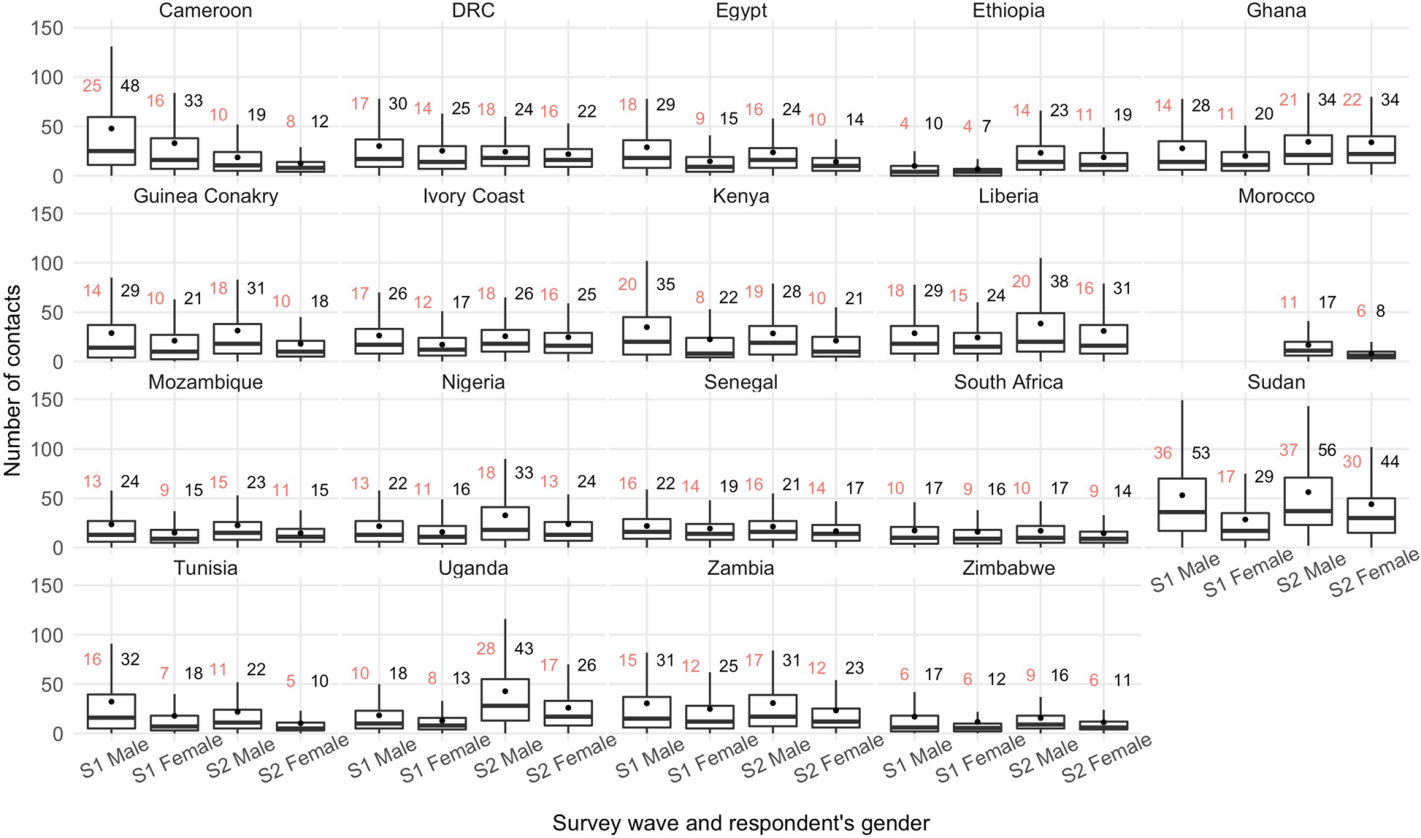
Median and mean contacts by country, survey wave, and respondent’s gender. Legend: red - median contacts, black - mean contacts, S1 - survey 1, S2 - survey 2

**Fig. 4:**
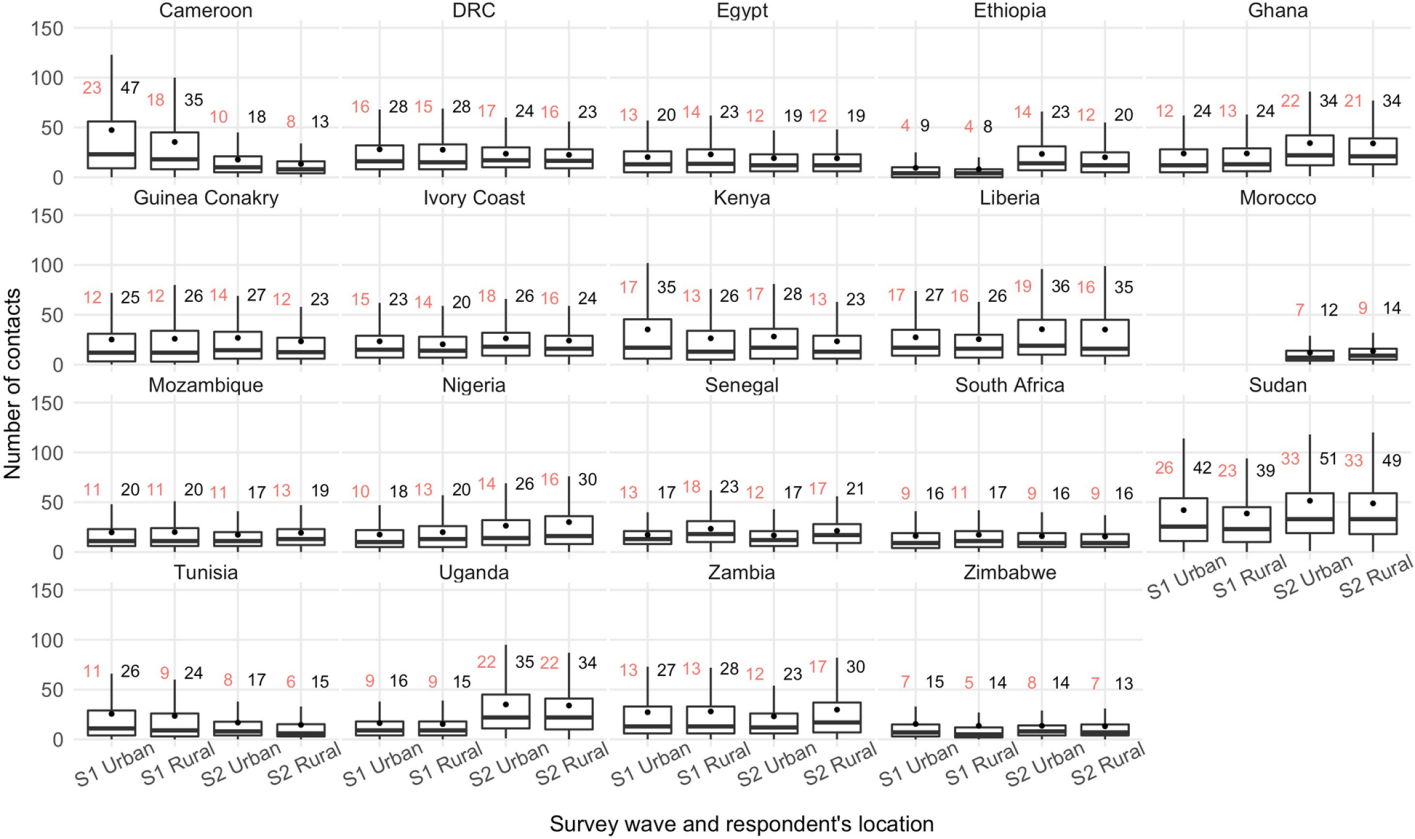
Median and mean contacts by country, survey wave, and respondent’s location. Legend: red - median contacts, black - mean contacts, S1 - survey 1, S2 - survey 2

The distribution of contacts by contactees’ age group (0–17, 18–55, 56–100) and contact setting (household, visitor, work or study, and other) was characterised (Figs. 1 and 2). In addition, we calculated the mean and median number of total daily contacts per country (Additional file 2: Table S4), according to respondents’ gender (male or female), residence location (urban or rural), household-head education status (no education, primary, secondary, tertiary, including post-graduate), household size (0–3, 4–6, or 7+), self-reported health (“Very bad”/“Bad”, “Fair”, or “Good”/ “Very good”), past or present SARS-CoV-2 infection status (yes, no, or uncertain), perceived COVID-19 self-reported infection risk (“Low”/“Very Low”, “Medium”, or “High”/“Very high”), the self-reported impact of COVID-19 on health (“Not at all”, “Somewhat”, “Very”, or “Extremely” seriously), mask ownership ("Yes" or "No") for surveys 1 and 2, and vaccine acceptance (“Definitely”, “Probably”, “Definitely not”, or “Probably not”) for survey 2 (Additional files 3, 4, 5, 6, 7, 8, 9, 10 and 11). A permutation test was performed for the main results to determine whether the differences in the mean or median number of daily contacts per respondent were significant at the 1% to 0.01% levels or were due to chance, using the country-specific samples for each survey (Additional file 2: Table S5). Entries with missing data on any of these variables were not included in the statistical analysis. Variables with notable or surprising differences are presented in Figs. 3 and 4, while the remainder are included as additional files.

**Fig. 5:**
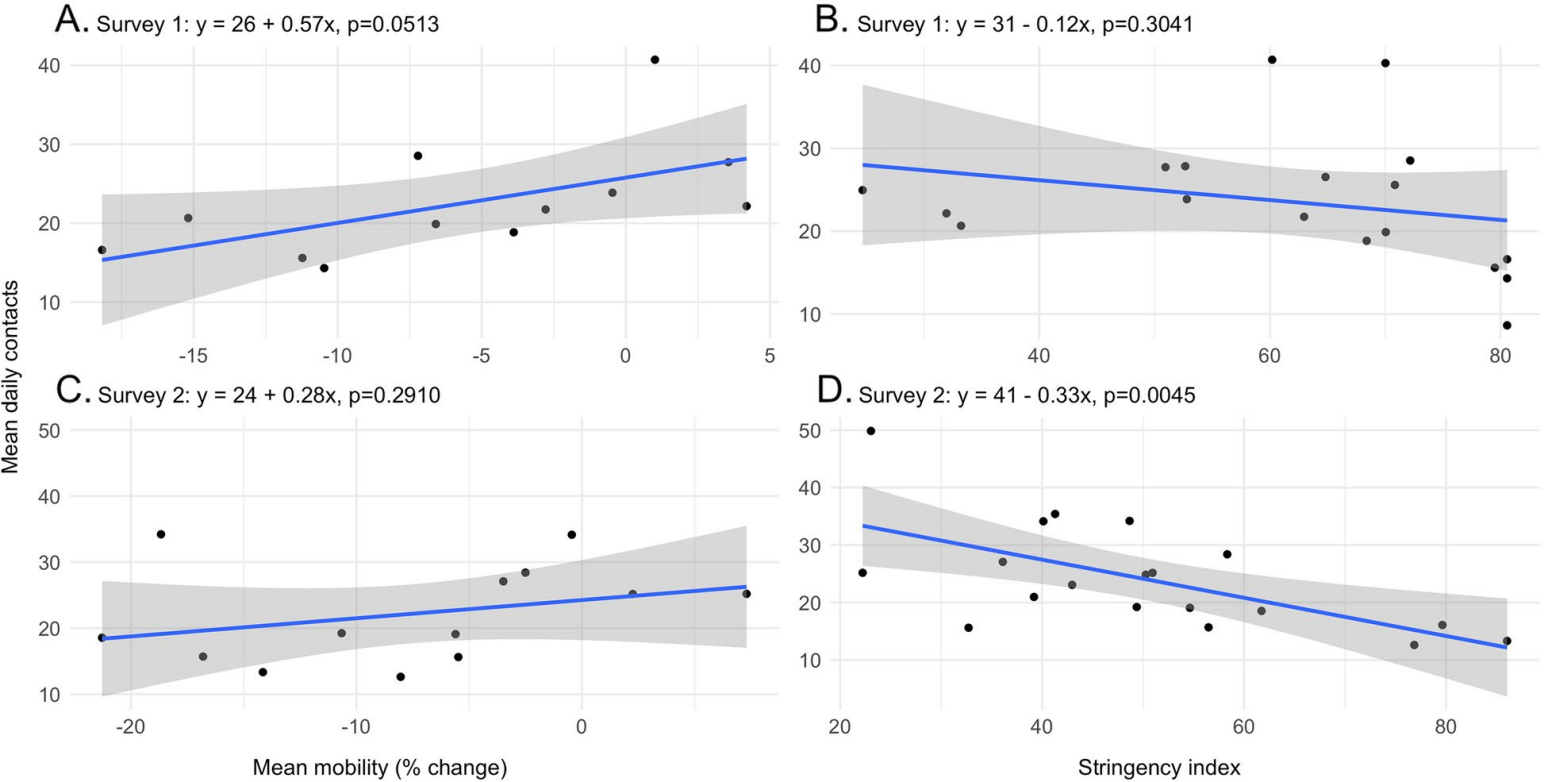
Relationship between median contacts and change in mobility and restrictions stringency. Legend: blue line - OLS fitted line, grey area - 95% confidence

Finally, mean and median daily contacts were regressed against the change in mobility from pre-pandemic levels and the stringency of restrictions (Fig. 5 and Additional file 12: Fig. S10). For the change in mobility, publicly available national-level Google Community Mobility Reports were used, which show mobility trends in different locations (retail and recreation, groceries and pharmacies, parks, transit stations, workplaces, and residential) [27]. The dependent variable was defined as the mean or median reported number of contacts in the surveyed countries. The independent variable was defined as the mean change in mobility recorded across all locations during each survey period. No data were available for the Democratic Republic of Congo (DRC), Ethiopia, Guinea, Liberia, Sudan, and Tunisia. The Oxford Government Response Stringency Index is a composite index of the stringency scores of eight indicators (school closing, workplace closing, cancellation of public events, restrictions on gatherings, closure of public transport, stay-at-home requirements, restrictions on movement, and restrictions on international travel) (Additional file 2: Table S6) [28]. The independent variable for this analysis was defined as the mean absolute stringency index reported during each survey period. The estimated model intercepts and coefficients are reported with their standard errors and p-values.

### Ethics

Participation was voluntary, and data were anonymised. Interviewers explained the purpose of the study in the respondent’s preferred language, and informed consent was obtained before participation. Ipsos Mori, a market research company based in the United Kingdom (UK), conducted the surveys. Ethical approvals from the London School of Hygiene and Tropical Medicines (LSHTM) were obtained for the survey (Ref: 22486-1) and current analysis (Ref: 25945) as well as from all national ethics committees.

## Results

### Respondent characteristics

Twenty-three thousand nine hundred forty respondents and 563,976 contacts were analysed across the 18 countries for survey 1 and 25,640 respondents and 607,142 contacts across the 19 countries for survey 2. The percentage of people who agreed to participate out of all people randomly called (i.e. the participation rate) was on average 41% for survey 1 and 50% for survey 2 (Additional file 2: Table S7). The lowest participation rate was recorded in Morocco for survey 2 of 11% and the highest for Sudan in survey 2 of 83%. Most countries had a participation rate of at least 30%.

For survey 1, the median age of respondents ranged between 26 in Guinea and Zambia and 41 in Tunisia; between 42% (Guinea) and 52% (Sudan) were female (Table 1). For survey 2, the median age ranged between 26 in Guinea and Sudan and 41 in Morocco and Tunisia; between 45% (Kenya) and 53% (South Africa) were female (Additional file 2: Table S3). The sampled populations followed a similar age structure across countries in both surveys. Considering the absence of the 0–17 age group, adults aged over 60 were under-sampled in all countries for both surveys (Additional file 2: Table S2) [26]. Female respondents were represented well, when compared to population estimates (Additional file 2: Table S3) [26]. Between 24% (Uganda) and 58% (South Africa) resided in urban areas in survey 1 and between 21% (Uganda) and 67% (Tunisia) in survey 2. Compared to the World Urbanisation Prospects estimates (Additional file 2: Table S3) [29], the rural population was under-sampled in DRC, Ethiopia, Guinea, and Sudan and oversampled in Cameroon and South Africa in survey 1. The rural population was somewhat under-sampled in DRC and Kenya and oversampled in Nigeria and South Africa in survey 2. For the remaining countries, the rural and urban populations were represented well.

The median household size ranged between 5 and 9 in survey 1 (Table 1) and between 4 and 10 in survey 2 (Additional file 2: Table S1). In most countries, respondents had primary, secondary or tertiary education representing between 20% and 55% of the survey 1 respondents. Similar was the case for survey 2, except in Morocco and Guinea where over 35% of the respondents had no education (Additional file 2: Table S1). In Survey 1, Guinea, Kenya, Liberia, Mozambique, and Uganda, approximately 30% of respondents had an income of $0–$100. In Zimbabwe, these were 83% of the respondents. In Cameroon, DRC, Egypt, Ethiopia, Ghana, Ivory Coast, Nigeria, Sudan, Tunisia, and Zambia, those with an income of $101–$500 USD represented 40% or more of the sample. These in turn represented 35% and 28% of the samples in Senegal and South Africa, respectively. 20% of the observations for Cameroon, Mozambique, Nigeria, South Africa, and Senegal were missing income data. Reported income was not comparable across countries for survey 2 as it was recorded in the local currency.

### Contact patterns

The majority of contacts in each country occurred with contactees aged 18 to 55, representing 50% or over of total contacts for both surveys (Fig. 1). Between 20% and 40% of all contacts were with contactees aged 0 to 17. A similar trend was observed for both surveys. Contacts with those over 55 were less than 20% of total contacts in most countries. For both surveys, most contacts occurred in a household or work, school, or university settings (Fig. 2). Household contacts accounted for between 20% and 40% of all contacts. Contacts in work, school or university settings accounted for between 30% and 40%+ of all contacts. The smallest number of contacts occurred in the remaining two settings (other and visitors), except in Kenya, Mozambique, Sudan, and Zimbabwe, where other contacts accounted for the majority in survey 1. Most countries displayed a similar pattern of contacts across survey rounds (e.g. Cameroon, DRC, Egypt, Ghana, Guinea, Ivory Coast, Liberia, Senegal, South Africa, Sudan, and Zambia). However, there were some notable changes across survey rounds where most contacts occurred: from a household to a work/study setting in Ethiopia, Nigeria, Tunisia, and Uganda and from other to households in Mozambique and Zimbabwe.

For survey 1, the number of mean daily reported contacts per respondent varied greatly between countries ranging between 9 (SD=16, median = 4, IQR = 8) in Ethiopia and 41 (SD=56, median = 20, IQR = 41) in Cameroon (Additional file 2: Table S4). For survey 2, mean contacts ranged between 13 in Morocco (SD=15, median = 8, IQR = 11) and in Zimbabwe (SD=20, median = 7, IQR = 10) and 50 in Sudan (SD=53, median = 33, IQR = 40).

There was strong evidence to suggest that mean contacts increased between the surveys in Ethiopia (from 9 to 21), Ghana (from 24 to 34), Liberia (from 27 to 35), Nigeria (from 19 to 28), Sudan (from 40 to 50), and Uganda (from 16 to 34) (*p*-value < 0.0001). For these, the increase in the median number of contacts was also significant for all but Liberia. Mean contacts decreased from 41 to 16 in Cameroon, 28 to 23 in DRC, and 25 to 16 in Tunisia (*p*-value < 0.0001). Of these, the decrease in the median number of contacts was significant for Cameroon and Tunisia at this significance level. This was consistent with changes in the stringency of restrictions (Additional file 2: Table S6), except for Cameroon and DRC.

In general, male respondents reported more mean or median contants than female respondents (Fig. 3 and Additional file 2: Table S5). In survey 1, male respondents reported significantly more mean contacts than female respondents in Cameroon (48 vs 33), Egypt (29 vs 15), Ethiopia (10 vs 7), Ghana (28 vs 20), Ivory Coast (26 vs 17), Kenya (35 vs 22), Mozambique (24 vs 15), Nigeria (22 vs 16), Sudan (53 vs 29), Tunisia (32 vs 18), and Uganda (18 vs 13) (*p*<0.0001). Only in South Africa, no significant difference in the number of mean or median contacts was observed between the two genders. The overall trend was similar but no significant differences were observed for Ghana (mean contacts for both genders of 34) and Ivory Coast (26 vs 25) in survey 2. Male respondents in Morocco had double the contacts of female respondents (17 vs 8, *p*<0.0001) in survey 2. The differences in the median reported contacts for both genders were less significant in comparison, but followed the same trend.

The variation in the mean and median contacts by respondent’s location was less pronounced compared to that by gender (Fig. 4 and Additional file 2: Table S5). However, respondents in urban locations had more contacts on average than those in rural locations in Cameroon (survey 1: 47 vs 35; survey 2: 18 vs 13, *p*<0.0001) and Kenya (survey 1: 35 vs 26; survey 2: 28 vs 23, *p*<0.01). The opposite was the case in Senegal (survey 1: 17 vs 23; survey 2: 17 vs 21, *p*<0.0001) and Zambia (survey 2: 23 vs 30, *p*<0.001).

Variations in the mean and median contacts by different demographic characteristics or COVID-19 perceptions over the two surveys are further reported in Additional files 3, 4, 5, 6, 7, 8, 9, 10 and 11. There were no substantial differences across countries in the number of contacts by the household head education level (Additional file 3: Fig. S1), the respondent’s self-reported general health (Additional file 4: Fig. S2), the respondent’s past SARS-CoV-2 infection status (Additional file 5: Fig. S3), vaccine acceptance in survey 2 (Additional file 6: Fig. S4), or possession of a mask (Additional file 7: Fig. S5). Generally, respondents living in households of 7 or more members reported more non-household contacts (work/study, visitor, or other) than those in smaller households (e.g. of 1–3 or 4–6 members) (Additional file 8: Fig. S6). In some countries (e.g. Cameroon, DRC, Sudan), this applied to all contacts (Additional file 9: Fig. S7). Somewhat surprisingly, we did not find consistent differences across countries and waves in the mean number of contacts between respondents with low and high levels of perceived risk of catching SARS-CoV-2 (Additional file 10: Fig. S8), and perceived risk to health (Additional file 11: Fig. S9).

### Mean daily contacts, mobility change, and stringency index

The relationship between mean daily contacts and mobility change and stringency index follows the expected pattern across survey waves (Fig. 5). Mean daily contacts increase as mobility increases and decline as the stringency of restrictions increases. In particular, an increase of one percentage point in mobility is associated with an increase of 0.57 (standard errors (SE): 0.26, *p*=0.0513) contacts for survey 1 and 0.28 (SE: 0.25, *p*=0.2910) for survey 2, though neither are significant at the 5% level. A decrease of one unit in the stringency index was associated with a decrease of 0.12 (SE: 0.11, *p*=0.3041) contacts for survey 1 and 0.33 (SE: 0.10, *p*=0.0045) for survey 2, significant at the 5% level. A similar relationship is observed between median daily contacts and mobility change and the stringency index (Additional file 12: Fig. S10). However, the magnitude of the coefficient on mobility and stringency and their standard errors decreased compared to when mean contacts were used. A potential explanation for this is that using the meadian estimate instead of the mean decreases the variation in observations in the contacts by country. This in turn leads to a more significant but potentially diluted coefficient estimate.

## Discussion

These data show that social contacts differ markedly between and within countries. We also find an inverse relationship between the stringency of restrictions and mean contacts, suggesting that the implementation of NPIs follows their intent. We find that contacts were the lowest in Ethiopia (survey 1) and Morocco and Zimbabwe (survey 2), and the highest in Cameroon (survey 1) and Sudan (survey 2). The majority of contacts occurred in the household or work/study settings for both surveys. Most contacts occurred with people of working age (18–55), reflecting the generally higher social interactions associated with participating in economic activities. On average, men had more contacts than women and, in most countries, mean contacts did not differ by the respondent’s area of residence, except in Cameroon and Kenya (where respondents in urban locations reported more contacts than those in rural) and in Senegal and Zambia (where the opposite was observed).

Notably, considering that the surveys were conducted when contacts were discouraged, the reported mean number of contacts is consistently higher compared to previous estimates, for the countries where prior contact data are available [13, 15, 16, 18, 19, 21, 22]. The median number of reported contacts is closer to the past mean estimates. This could be due to a number of reasons. Firstly, while the respondent sample is close to nationally representative, respondents of working age (18–55) are slightly oversampled, potentially leading to a higher number of contacts. Additionally, many other studies report primarily on rural or semi/peri-urban communities or selected regions [13, 16, 18, 19, 21, 22], making direct comparisons challenging. Finally, variations in the surveys’ methodologies could also be driving the observed differences—in the current study contacts were estimated rather than listed leading to reduced precision and potentially increased variability.

Other studies have also found that men have more contacts than women, reflecting gender differences in economic activities outside the home in some communities [15, 30]. The finding that Senegalese respondents in urban areas make less contacts than in rural areas is consistent with findings of a prior Senegalese study on face-to-face contacts [19]. Surprisingly (and in contrast to our findings), available contact studies in Kenya, South Africa, and Zimbabwe report more contacts for rural rather than urban areas [14, 16, 21, 22]. This could be due to differences in the sampled populations and/or the definition of a contact.

We find that mean contacts increased between the two time points in Ethiopia, Ghana, Liberia, Nigeria, Sudan, and Uganda and decreased in Cameroon, DRC, and Tunisia, reflecting the opposite change in the restrictions between the two time points in all countries but Cameroon and DRC. We cannot exclude external factors that may confound this relationship. While the Cameroon and DRC findings are surprising, this could be due to, among many reasons, chance, poor quality stringency data, or because people are not responding to the restrictions.

There was a lack of significant difference in the number of contacts according to respondents’ perception of the COVID-19 risk to health in both surveys. This contrasts prior studies in high-income settings—a study in the UK reported fewer mean contacts for those who agreed that COVID-19 would have a high impact on their health than those who did not agree [31]. However, risk perceptions might impact contact patterns differently in low-and-middle-income countries. For example, a survey in communities of Tamil Nadu, India found that the majority of respondents perceived low level of risk of contracting COVID-19, whereby more common concerns were related to loss of income, inability to travel, or getting sick [32]. Other studies also support the finding that most contacts occur in non-household settings [13, 18] and, when tighter restrictions are in place such as a national lockdown, the proportion of household contacts is higher than in other contact settings [33]. More contacts of respondents in larger households than smaller ones have also been reported elsewhere [12].

There are some methodological limitations of this study. Firstly, the contact surveys in each country were conducted retrospectively via structured telephone interviews, prone to responder recall bias and fatigue. These may have led to respondents omitting contacts or rounding down or up their number of reported contacts. An improved study design might use prospective contact diaries that record individual contacts, or structured questionnaires that aim to minimise recall bias by thoroughly guiding respondents through their day and listing all contacts [34]. Additionally, recent technological advances in wearable proximity sensors have enabled researchers to measure epidemiologically important close contacts in resource-limited settings [17]. This remains an important area for future research. However, such designs were not feasible for this study given that collecting contacts data was not the primary focus of the survey, but also because of the large sample size (over 1000 observations) across the 18 and 19 countries in surveys 1 and 2, respectively. The direction of the responder bias is unknown since contacts could be both rounded up or down and therefore the total contacts per country could be under or overestimated. Nevertheless, a strength of this survey, in addition to the large sample size, is that it captured a range of characteristics about the contacts and the respondents. Collecting data on multiple variables could have made a prospective design more burdensome and introduced a high rate of loss to follow-up.

In addition, participants were asked to report the number of social contacts they had, having been asked about COVID-19 transmission risk and attitudes, and we cannot rule out that social desirability bias may have affected responses. While the extent of this occurring is difficult to determine, such bias would result in respondents underreporting the number of contacts leading to overall lower average number of contacts recorded for a given country.

Thirdly, a prerequisite for participation in the telephone survey was access to a functioning telephone, connectivity, and electricity, which might be limited in some countries, particularly in rural areas. Indeed, comparing the proportion of rural and urban participants to the World Urbanisation Prospects database showed that the rural population was under-sampled in DRC, Ethiopia, Guinea, and Sudan in survey 1 and in DRC and Kenya in survey 2, which might affect the generalisability of the reported mean contacts in those countries.

Fourthly, the participation rate for the two surveys was satisfactory given the survey design of randomly digit-dialling a great number of people and given that the two samples remained balanced against the general population on observed characteristics such as gender and location. Thus, it is unlikely that this introduced a strong bias in the results. However, there may unobserved characteristics which drive people to participate and which may have introduced bias in the contacts data. If this was the case, the direction and magnitude of the bias remains unknown.

Regarding the regression analysis, this relies on a small sample size of 18–19 countries and in the case of mobility even fewer since no mobility data existed for DRC, Ethiopia, Guinea, Liberia, Sudan, and Tunisia. While the observed relationship followed the expected pattern (e.g. mean/median contacts increased with increasing mobility and decreased with increasing intensity of the restrictions across countries, albeit with weak significance), the analysis may have been underpowered to detect statistically significant patterns in the data and did not account for confounding factors. Nevertheless, the observed relationship validates that the collected data on contact patterns are of good quality.

Finally, google mobility data patterns over-represent urban areas given the limited access to mobile phone, Google maps, and internet connection in rural areas. While we found no significant differences between the number of contacts in rural compared to urban areas in most countries, the observed relationship between mobility (biased towards urban areas) and the number of mean contacts per country is likely weakened due to the inherently different sample characteristics. Nevertheless, this analysis illustrates an informative finding that there is a correlation between the number of contacts and mobility change.

## Conclusions

These are the first COVID-19 social contact data collected and mean daily contacts reported for the majority of studied countries (all but Kenya, Ethiopia, and South Africa). We found a high reported number of daily contacts in all countries as well as large variations in mean contacts across countries. We also found large variations by gender whereby men had on average more daily contacts than women. We found no consistent patterns in the variation of mean contacts by other characteristics across the two survey waves and countries. We found that increased stringency and decreased mobility were associated with a reduction in the number of contacts.

These findings can be useful for infectious disease modelling studies in low- and middle-income settings. In particular, we offer an empirical basis for assumptions around the expected number of contacts by country, urban/rural location, and gender. There are also some important learnings for policy makers and implementers involved in pandemic planning and response. For example, the stringency index had a relatively small coefficient and was weakly associated with the number of reported contacts per country, although the strength of the association improved between the two surveys. While control measures need to be appropriately implemented to ensure adherence, our study suggests they may have limited effects on reducing contacts in the countries studied. This highlights the need to develop locally appropriate social control measures that are implementable. It also stresses the necessary support for communities to help ensure adherance to individual measures (e.g. through the provision of resources such as improved risk communication strategies, infection, prevention and control materials, and clean water and soap for handwashing).

## Supplementary Information

**Additional file 1.** Interview questions.

**Additional file 2: Table S1.** Survey 2 Respondent Descriptives. **Table S2.** Population age structure. **Table S3.** Percentage female and rural population. **Table S4.** Mean, median, standard deviation, interquartile range (IQR) contacts in both surveys. **Table S5.** Permutation test results by main respondent characteristic and survey wave. **Table S6.** Oxford Stringency Index for surveys 1 and 2. **Table S7.** Percentage of people who agreed to participate out of total people called.

**Additional file 3: Fig. S1.** Contacts by household head education level.

**Additional file 4: Fig. S2.** Contacts by self-reported general health.

**Additional file 5: Fig. S3.** Contacts by past SARS-CoV-2 infection status.

**Additional file 6: Fig. S4.** Contacts by vaccine acceptance attitude (Survey 2 only).

**Additional file 7: Fig. S5.** Contacts by mask ownership.

**Additional file 8: Fig. S6.** Non-household contacts by household size.

**Additional file 9: Fig. S7.** Contacts by household size.

**Additional file 10: Fig. S8.** Contacts by perceived risk of catching SARS-CoV-2.

**Additional file 11: Fig. S9.** Contacts by perceived impact of COVID-19 on health.

**Additional file 12: Fig. S10.** Relationship between median contacts and change in mobility and restrictions stringency.

## Data Availability

Consent to make individual-level survey data was not obtained. However, aggregated results on contact characteristics that might be of interest are presented in the main body and additional material.

## Abbreviations

CNES: Comité National d'Éthique de la Sante
CNESVS: Comité National d’Éthique des Sciences de la Vie et de la Sante
COVID-19: Coronavirus disease
DRC: Democratic Republic of Congo
ERES: Excellence in Research Ethics and Science
IQR: Inter-quartile range
LMICs: Low- and middle-income countries
LSHTM: London School of Hygiene and Tropical Medicine
NPI: Non-pharmaceutical intervention
PERC: Partnership for Evidence-based Responses to COVID-19
RDD: Random digit dial
SARS-CoV-2: Severe acute respiratory syndrome coronavirus 2
SE: Standard errors
SD: Standard deviation
S1: Survey 1
S2: Survey 2
UK: United Kingdom
WPP: World Population Prospects
