# Supplementary material for: Characterising social contacts under COVID-19 control measures in Africa
Source: BMC Med. 2022 Oct 12;20:344. doi: 10.1186/s12916-022-02543-6 (PMC9553295; doi:10.1186/s12916-022-02543-6)
Supplement: Supplementary file 1 — Additional file 1. Interview questions. [file 12916_2022_2543_MOESM1_ESM.docx]

**INTRODUCTION AND CONSENT**

INTERVIEWER NAME:_________________________________

PHONE NUMBER CALLED ______________________________

**Greeting,**

Hello. My name is ____ and I am working for Ipsos, which is a private organization working on a survey to generate data on behalf of Africa CDC. The aim of this study is to understand people’s knowledge, perception and attitudes to the coronavirus and COVID-19.

You have been selected at random to participate in an important survey. All the Information you give us will be kept confidential. We will not ask your name.

The anonymized results may be shared with scientific experts or other organizations working with Africa CDC to help find ways to minimise the public health, social and economic impact of the virus. They may also be used in scientific publications.

Any answers you give us will be put together with answers from other people, and it will not be possible to identify anyone in the results.

The interview is completely voluntary, you have the right to participate or not, to stop at any time, and not answer any questions you don’t want to. The study doesn’t include anyone under 18 years of age. There are no right or wrong answers; we just want to find out your views. The interview will take up to 20 minutes to complete. Do you have time for me to ask you some questions now?

**Yes**, I agree to participate in this survey

**No**, I do not agree to participate in this survey (terminate)

*INTERVIEWER – IF THE PERSON SAYS NO, THEN THANK THEM FOR THEIR TIME AND END THE CONVERSATION. THIS SHOULD THEN BE RECORDED AS A* ***REFUSAL IN YOUR EXCEL FILE THAT LOGS ALL CALLS****.*

**S1a. SCREENER:**  **Are you 18 years or older?**

- 1. Yes (*Ask Question S1b*)
- 2. No – **THANK YOU FOR YOUR WILLINGNESS TO TALK TO ME TODAY BUT YOU ARE NOT ELIGIBLE TO TAKE THIS SURVEY**

**S1b. SCREENER: Do you agree to participate in this survey?**

- 1. Yes
- 2. No **THANK YOU FOR YOUR TIME**

**S1c. SCREENER: Do you mind telling us your permanent residential city/town/village?**

- 1. Insert city1/region
- 2. Insert city2/region
- 3. Insert city3/region
- 4. Insert city4/region
- 5. Insert city5/region

1. **KNOWLEDGE, MISINFORMATION & ATTITUDES**

ASK ALL

1. **Before now, had you heard of something called the Coronavirus or Covid-19?**
2. Yes – GO TO Q2 WITHOUT READING DESCRIPTION
3. No – READ DESCRIPTION, THEN GO TO Q2
4. Don’t know – READ DESCRIPTION, THEN GO TO Q2

DESCRIPTION TO BE READ TO THOSE WHO CODE 2 OR 3 AT Q1:

**Coronavirus is an infectious disease caused by a newly discovered virus.**

ASK ALL

1. **I’m going to read things people have said about coronavirus or Covid-19. Please tell me if you think each is definitely true, probably true, probably false, or definitely false. If you don’t know, say so.** (TREND W1)

RANDOMIZE ORDER OF STATEMENTS

|  | Definitely True | Probably True | Probably False | Definitely False | Don’t Know |
| --- | --- | --- | --- | --- | --- |
| **A).Washing hands helps prevent getting it** (TREND W1) | **1** | **2** | **3** | **4** | **9** |
| **B). People who have recovered from it should be avoided to prevent spreading it** (TREND W1) | **1** | **2** | **3** | **4** | **9** |
| **C). Infected people may not show symptoms for 5 to 14 days** (TREND W1) | **1** | **2** | **3** | **4** | **9** |
| **D). Wearing a face mask when around other people prevents the spread of it** |  |  |  |  |  |
| **E). It can be cured with herbal medicines** | **1** | **2** | **3** | **4** | **9** |
| **F). Foreigners are discrediting African medicines which could cure it** | **1** | **2** | **3** | **4** | **9** |
| **G). Foreigners are trying to test vaccines on us** | **1** | **2** | **3** | **4** | **9** |
| **H). Close contact with livestock and other animals is a risk for catching COVID-19** | **1** | **2** | **3** | **4** | **9** |

1. **I would now like to ask your opinion about face masks, which some people are wearing at the moment. To what extent do you agree or disagree with the following statements.**

RANDOMIZE ORDER OF STATEMENTS

|  | Strongly agree | Somewhat agree | Neither agree nor disagree | Somewhat disagree | Strongly disagree | Don’t know |
| --- | --- | --- | --- | --- | --- | --- |
| **When other people wear face masks near me, I think…** |  |  |  |  |  |  |
| **A)… they may be infected, and I should stay away from them** | 1 | 2 | 3 | 4 | 5 | 9 |
| **B)… they are being careful, and I appreciate that they are protecting others** | 1 | 2 | 3 | 4 | 5 | 9 |
| **C)… they are being foolish, because this is unlikely to protect anyone** | 1 | 2 | 3 | 4 | 5 | 9 |

1. **Do you have a face mask of any kind that is ready for you to wear?**
2. Yes – ASK Q5
3. No – GO TO Q6
4. Don’t know/refused – GO TO Q7

ASK WHO SAY “YES” (CODE 1 AT Q4)

1. **What type of face mask do you have?** (INTERVIEWER: DO NOT READ OUT. MULTICODE OK IF MORE THAN ONE TYPE USED)
2. Surgical mask (INTERVIEWER: THESE FIT LOOSELY ACROSS THE NOSE AND MOUTH. THEY ARE OFTEN BLUE BUT CAN ALSO BE WHITE OR OTHER COLOURS).
3. Respirator/filtering facepiece/N95/N99/FFP1,2,3 (INTERVIEWER: THESE FIT TIGHTLY AROUND THE FACE AND HAVE A FILTER)
4. Dust mask (INTERVIEWER: THESE LOOK SIMILAR TO RESPIRATORS BUT DON’T HAVE A FILTER, THEY ARE SOMETIMES USED FOR DIY/HOME IMPROVEMENT PROJECTS)
5. Washable face covering (purchased) (INTERVIEWER: THESE ARE OFTEN MADE OF SPONGE, WITH A MATERIAL COVERING)
6. Cloth face covering (purchased)
7. Cloth face covering (home-made)
8. Face visor
9. Other (specify)
10. Don’t know

ASK ALL WHO SAY “NO” (CODE 2 AT Q4)

1. **We are trying to better understand why some people have a face mask and some don’t. You say that you currently don’t have a mask. Why is that?** (INTERVIEWER: DO NOT READ OUT. MULTICODE OK).
2. Don’t know how/where to get one
3. Don’t have time to get/make one
4. Don’t know what type to get/how to make one
5. Shops/markets sold out (to buy mask/to buy materials to make face covering)
6. Shops/markets closed (to buy mask/to buy materials to make face covering)
7. Can’t get to shops/markets/too far away (to buy mask/to buy materials to make face covering)
8. Can’t afford it (to buy mask/to buy materials to make face covering)
9. Critical illness/breathing difficulties/mobility difficulties makes it hard to wear one
10. I used to have one, but it’s broken
11. I used to have one, but it was lost/stolen
12. I used to have one, but gave it to someone else
13. I don’t think they are necessary/don’t help protect/prevent spread
14. I don’t like them/they are uncomfortable
15. I am worried people will think I am infected if I wear one
16. I don’t need one/don’t go out/don’t mix with others outside my household
17. They are not mandatory
18. Other (specify)
19. Don’t know
20. **RISK PERCEPTIONS**

ASK ALL

1. **To what extent do you agree or disagree with the following statements?**

|  | Strongly agree | Somewhat agree | Neither agree nor disagree | Somewhat disagree | Strongly disagree | Don’t know |
| --- | --- | --- | --- | --- | --- | --- |
| **A). The coronavirus will affect very many people in the country I’m currently living in** | 1 | 2 | 3 | 4 | 5 | 9 |
| **B). I am confident in my ability to follow information and restrictions given by the government to reduce my risk of getting COVID-19** | 1 | 2 | 3 | 4 | 5 | 9 |

1. **Please indicate what you think your level of risk of catching coronavirus or Covid-19 is:**

(TREND W1)

1. Very high
2. High
3. Medium
4. Low
5. Very Low
6. Don’t know
7. **If you were infected by coronavirus or Covid-19, how seriously do you think it would affect your health?** (TREND W1)

1. Not at all seriously
2. Somewhat seriously
3. Very seriously
4. Extremely seriously
5. Don’t know / no response
6. **SOCIAL COHESION**
7. **There are public health guidelines recommended to restrict the spread of coronavirus. These include maintaining a minimum distance from people you are not in contact with regularly and limiting the number of people who can gather together.**

**Which is closer to your point of view about taking these recommended actions?** (READ OUT STATEMENTS A TO C IN TURN)

| A) | **Taking these actions will protect me from getting infected with COVID-19…** | **OR** | **...taking these actions will have no impact on the likelihood of getting infected** | Don’t know |
| --- | --- | --- | --- | --- |
|  | | | | |
| B) | **Taking these actions will protect other members of my household from getting infected with COVID-19…** | **OR** | **… taking these actions will have no impact on the likelihood of getting infected** | Don’t know |
|  | | | | |
| C) | **Taking these actions will protect others I come in contact with from getting infected with COVID-19** | **OR** | **… taking these actions will have no impact on the likelihood of getting infected** | Don’t know |

1. **TRUST & CONFIDENCE IN GOVERNMENT RESPONSE**
2. **How satisfied are you with your (COUNTRY NAME) government’s response to coronavirus or Covid-19?** (TREND W1)
3. Very satisfied
4. Somewhat satisfied
5. Somewhat dissatisfied
6. Very dissatisfied
7. Don’t know / not sure
8. **To what extent, if at all, do you trust each of the following individuals and organizations’ handling of the coronavirus in [COUNTRY]?**

|  | **A great deal** | **A fair amount** | **Not very much** | **Not at all** | Don’t know | Not heard of organization | Not Applicable |
| --- | --- | --- | --- | --- | --- | --- | --- |
| **A) Your own family doctor** | 1 | 2 | 3 | 4 | 5 | 6 | 7 |
| **B) Traditional healers** | 1 | 2 | 3 | 4 | 5 | 6 | 7 |
| **C) Your Employer (if applicable)** | 1 | 2 | 3 | 4 | 5 | 6 | 7 |
| **D) Media** | 1 | 2 | 3 | 4 | 5 | 6 | 7 |
| **E) Hospitals/ health centers** | 1 | 2 | 3 | 4 | 5 | 6 | 7 |
| **F) Ministry of Health** (TAILOR TO EACH COUNTRY) | 1 | 2 | 3 | 4 | 5 | 6 | 7 |
| **G) Medical professional associations (e.g….** (ADAPT TO LOCAL CONTEXTS)) | 1 | 2 | 3 | 4 | 5 | 6 | 7 |
| **H) Schools** | 1 | 2 | 3 | 4 | 5 | 6 | 7 |
| **I) Community health workers** | 1 | 2 | 3 | 4 | 5 | 6 | 7 |
| **J) Police** | 1 | 2 | 3 | 4 | 5 | 6 | 7 |
| **K) Army/military** | 1 | 2 | 3 | 4 | 5 | 6 | 7 |
| **L) Religious institutions** | 1 | 2 | 3 | 4 | 5 | 6 | 7 |
| **M) The President** [ONLY ASK IN COUNTRIES WITH A PRESIDENT] | 1 | 2 | 3 | 4 | 5 | 6 | 7 |
| **N) Africa Center for Disease Control (Africa CDC)** | 1 | 2 | 3 | 4 | 5 | 6 | 7 |
| **O) World Health Organization (WHO)** | 1 | 2 | 3 | 4 | 5 | 6 | 7 |
| **P) UNICEF** | 1 | 2 | 3 | 4 | 5 | 6 | 7 |

1. **PHSM Support**
2. **Over the past seven days, to what extent do each of the following describe you personally? Would you say it completely applies, most applies, sometimes applies or does not apply to you?**

|  | **Completely applies** | **Mostly applies** | **Sometimes apples** | **Does not apply to me** |
| --- | --- | --- | --- | --- |
| **A) Washing my hands with soap or using hand sanitizer many times per day** | **1** | **2** | **3** | **4** |
| **B) Avoiding handshakes and physical greetings** | **1** | **2** | **3** | **4** |
| **C) Staying home instead of going to work, school or other regular activities** | **1** | **2** | **3** | **4** |
| **D) Reducing the number of times I go to the market or grocery store** | **1** | **2** | **3** | **4** |
| **E). Avoiding the church/mosque** | **1** | **2** | **3** | **4** |
| **F) Avoiding public gatherings and places of entertainment** | **1** | **2** | **3** | **4** |
| **G) Wearing a face mask in public when near others** | **1** | **2** | **3** | **4** |

**I would now like to ask you about measures some governments in Africa have implemented in response to the coronavirus.**

1. **Thinking of the last four weeks, was asking people to do each of the following absolutely necessary, somewhat necessary or not really necessary to limiting the spread of COVID-19 in [COUNTRY]?**

|  | Absolutely necessary | Somewhat necessary | Not really necessary | Don’t know |
| --- | --- | --- | --- | --- |
| **A) ...wash their hands with soap or use hand sanitizer more often than they used to** | 1 | 2 | 3 | 9 |
| **B) ...avoid handshakes and physical greetings** | 1 | 2 | 3 | 9 |
| **C) ... staying home instead of going to work, school or other regular activities** | 1 | 2 | 3 | 9 |
| **D) ...reduce the number of times people go to the market or grocery store** | 1 | 2 | 3 | 9 |
| **E). ...stop going to the church/mosque** | 1 | 2 | 3 | 9 |
| **F) … stop joining public gatherings and places of entertainment** | 1 | 2 | 3 | 9 |
| **G) … wear a face mask in public when near others** | 1 | 2 | 3 | 9 |

1. **To what extent to you agree or disagree with the following statements?**

|  | Strongly agree | Somewhat agree | Neither agree nor disagree | Somewhat disagree | Strongly disagree | Don’t know |
| --- | --- | --- | --- | --- | --- | --- |
| **A). Thinking about resuming normal activities after the lockdown makes me feel very anxious** | **1** | **2** | **3** | **4** | **5** | **9** |
| **B) I would feel comfortable taking public transportation such as local buses or trains if it was not too busy** |  |  |  |  |  |  |
| **C) It will be at least six months before the coronavirus is contained** |  |  |  |  |  |  |

1. **Some people say that loosening restrictions now puts too many people at risk of contracting COVID-19 and we need to wait at least a few more weeks. Other people say that the health risk is minimal if people follow social distancing rules and we need to get the economy moving again. Which is closer to your point of view?**
2. **Loosening restrictions now puts too many people at risk of contracting COVID-19 and we need to wait at least a few more weeks**
3. **The health risk is minimal if people follow social distancing rules and we need to get the economy moving again. Which is closer to your point of view?**
4. Don’t know
5. **Burden**
6. **In the past 7 days, how many days have you or someone in your household experienced any of the following?**

RANDOMIZE ORDER OF STATEMENTS

|  | Number of days |
| --- | --- |
| **a)…had difficulties in going to food markets due to mobility restrictions imposed by the government** |  |
| **b)…had difficulties in buying food due to most food markets being closed?** |  |
| **c)…been unable to buy the amount of food you usually buy because of shortages in the markets you buy from?** |  |
| **d)…been unable to buy the amount of food you usually buy because the price was too high?** |  |
| **e)…been unable to buy the amount of food you usually buy because your income has dropped?** |  |

1. **How does the amount of money you made in the past 7 days compare to the amount you made this time last year? Is it...** (READ OUT)

1) **Bigger**
 2) **Stayed the same**
 3) **Smaller**
 4) Don't make any (DO NOT READ)
 9) Don't know (DO NOT READ)

1. **Since the crisis began, have you had a change in your hours spent on unpaid work, such as childcare, care of the elderly and household work? Is it…**
   1. **More hours**
   2. **Less hours**
   3. **No change in hours**

9) Don’t know (DO NOT READ)

1. **Have you received any food, cash or other support from the government, in the past month that you do NOT usually receive? If so, which type of support?** (MULTIPLE RESPONSES POSSIBLE FOR YES)
2. No, none
3. Yes – food
4. Yes – cash
5. Yes – Personal Protective Equipment (PPEs) e.g. masks
6. Yes – hygiene supplies e.g. soap/chlorine/veronica buckets
7. Yes –free/subsidized services e.g. water/electricity/fuel
8. Yes – Other (specify)
9. **Have you or any other person in your household delayed, skipped or been unable to complete health care visits since the COVID-19 crisis?**
10. Yes… ASK Q22
11. No – have not delayed/skipped or been unable to complete health care visits… GO TO Q24
12. No – have not needed health care visits… GO TO Q24
13. Don’t know… GO TO Q24

ASK ALL WHO SAY “YES” (CODE 1, Q21)

1. **Why have you or another person in your household delayed, skipped or been unable to complete health care visits since the COVID-19 crisis?** (OPEN END WITH PRECODES, DO NOT READ RESPONSES, CODE ALL MENTIONS)
2. Not had time generally
3. Not had time due to childcare/schools being closed
4. Not had time due to caring for sick household member (s)
5. My health care issue did not seem urgent/wanted to free up facilities for those who may need it more
6. Health care facilities have been too busy/couldn’t get an appointment/they couldn’t see me/not enough health care workers
7. Health care facilities have been closed
8. Couldn’t afford it
9. Health care facility too far away/nobody to take me/public transport not working
10. Couldn’t get to health care facility due to lockdown/curfew
11. Self-isolating with suspected COVID-19 symptoms
12. Worried about going out/risk of catching coronavirus at health facility
13. Other (specify)
14. Don’t know

ASK ALL WHO SAY “YES” (CODE 1, Q21)

1. **And do you mind if I ask what those delayed, missed or not completed health care visits were for?** (OPEN END WITH PRE-CODES, DO NOT READ RESPONSES, CODE ALL MENTIONS)
2. General/routine check up
3. Perinatal care/problems with pregnancy/problems following a recent birth
4. Antenatal care
5. Care for children aged under 5
6. Family planning
7. Vaccinations
8. Cancer treatment
9. Cardio-vascular issues/heart problems/stroke/angina/high blood pressure
10. Diabetes
11. Respiratory problems/asthma
12. Suspected coronavirus symptoms
13. Mental health issues/anxiety/depression
14. Neurological disorders (e.g., stroke …)
15. Suspected broken bones/sprains
16. Malaria
17. Tuberculosis
18. HIV treatment
19. Other (specify)
20. Refused

ASK ALL

1. **Has the COVID-19 crisis made it more difficult to obtain medications you need for you or your household or has it had no impact? If you do not need medications, please say so.**
2. Much more difficult
3. A bit more difficult
4. No impact
5. I/my household do not need medicines
6. Don’t know
7. Refused
8. **How often have you experienced the following over the last 2 weeks?**

|  | **Not at all** | **Rare, less than a day or two** | **Several days** | **More than 7 days** | **Nearly every day over the last 2 weeks** | Don’t know/ refused |
| --- | --- | --- | --- | --- | --- | --- |
| **A). I felt dizzy, lightheaded, or faint, when I read or listened to news about the coronavirus** | 1 | 2 | 3 | 4 | 5 | 9 |
| **B). I had trouble falling or staying asleep because I was thinking about the coronavirus** | 1 | 2 | 3 | 4 | 5 | 9 |
| **C). I felt paralyzed or frozen when I thought about or was exposed to information about the coronavirus** | 1 | 2 | 3 | 4 | 5 | 9 |
| **D). I lost interest in eating when I thought about or was exposed to information about the coronavirus** | 1 | 2 | 3 | 4 | 5 | 9 |
| **E). I felt nauseous or had stomach problems when I thought about or was exposed to information about the coronavirus** | 1 | 2 | 3 | 4 | 5 | 9 |

1. **Over the last 24 hours how many people have you had contact with? A contact should be anyone with whom you exchanged at least a few words and were close enough to not need to raise your voice or you had direct physical contact with (including handshaking, kissing or other contact) in the following settings…?**

|  | Number of people – Aged under 18 | Number of people – Aged 18-55 | Number of people – Aged Over 55 |
| --- | --- | --- | --- |
| **a) People within your household (INTERVIEWER: This means people within the respondents’ household, not visitors)** | 1 | 2 | 3 |
| **b) People visiting your household or compound** | 1 | 2 | 3 |
| **c) People at work, school or university** | 1 | 2 | 3 |
| **d) People in other places** | 1 | 2 | 3 |

1. **THERE IS NO Q27**
2. **Do you have, or have you had, the coronavirus or COVID-19?**
3. Yes, confirmed by health care professional/tested positive
4. Yes, I’m sure I have but not confirmed
5. I’m not sure, but I think so
6. I’m not sure, but I don’t think so
7. No
8. Don’t know
9. **Do any other members of your household have, or have they had, the coronavirus or COVID-19?**
10. Yes, confirmed by health care professional/tested positive
11. Yes, I’m sure they have but not confirmed
12. I’m not sure, but I think so
13. I’m not sure, but I don’t think so
14. No
15. Don’t know

ASK ALL WHO SAY THEY HAVE HAD COVID-19 OR THINK SO (Q28, CODES 1, 2, 3)

1. **You said that you have been infected by COVID-19. What, if anything did you do about it?**

ASK ALL WHO SAY OTHER MEMBERS OF THEIR HOUSEHOLD HAVE HAD COVID-19 OR THINK SO (Q29, CODES 1, 2, 3)

1. **You said that another member of your household has been infected by COVID-19. What, if anything did you /they do about it?**

DO NOT READ OUT. MULTICODE OK APART FROM DON’T KNOW/NOTHING.

|  | Q30 – RESPONDENT | Q31– OTHER HOUSEHOLD MEMBER |
| --- | --- | --- |
| 1) Contact a nearby health facility |  |  |
| 2) Go/take the person to a hospital |  |  |
| 3) Contact the national helpline for support |  |  |
| 4) Inform a community health worker |  |  |
| 5) Isolate myself or the infected person |  |  |
| 6) Buy medication from a nearby pharmacy |  |  |
| 7) Apply home remedies |  |  |
| 8) Others(specify) |  |  |
| 9). Nothing |  |  |
| 10).Don’t know |  |  |

1. **Socio-demographics**

ASK ALL

Finally, a few questions for statistical purposes only

1. **What is your age?** RECORD:___
2. **Thinking now about the person who you consider to be the head of your household, what is the highest level of education they have completed?** INTERVIEWER: ASK RESPONDENT FOR BEST ESTIMATE IF RESPONDENT NOT SURE
3. No formal education
4. Incomplete primary school
5. Completed primary school
6. Incomplete secondary school
7. Completed secondary school
8. Some university / College of education / technical or vocational school
9. University (first) degree
10. Post-graduate degree
11. Don’t know / refused
12. **Including yourself, how many people are there in your household?** RECORD:___
13. **And, again including yourself, how many people in your household worked for one hour or more for pay in the last 7 days?** RECORD:___
14. **Would you have a separate room in your home to keep someone isolated if they are sick?**
15. Yes
16. No
17. Don’t know / no response
18. **What kind of water source do you have at home?** (OPEN END WITH PRECODES)
19. Running water in house
20. Communal tap/well
21. Boreholes
22. Protected springs
23. Truck
24. Bottled
25. Don’t know
26. **ETHNIC GROUP QUESTION, TAILORED TO COUNTRY.**
27. Categories tailored by country
28. Prefer not to answer
29. **If you think about your total monthly family income, which of these categories does it fit into?**

| Amount in USD | Local currency equivalent |
| --- | --- |
|  |  |
| 0 to 100 USD |  |
| 101 to 200 USD |  |
| 201 to 500 USD |  |
| 501 to 1,000 USD |  |
| 1,001 to 2,000 USD |  |
| 2,001 to 5,000 USD |  |
| Over 5,000 USD |  |
| Refused |  |
| Don’t know |  |

1. **How is your health in general, is it...** (INTERVIEWER READ OUT)
2. **Very good**
3. **Good**
4. **Fair**
5. **Bad**
6. **Very bad**
7. Refused (DO NOT READ OUT)
8. Don’t understand the question (DO NOT READ OUT)
9. Don’t know (DO NOT READ OUT)
10. **Do you have any longstanding illness or health problem?**

(INTERVIEWER: LONGSTANDING MEANS ONE THAT LASTS (OR WILL LAST) 6 MONTHS OR MORE, OR THAT IT REGULARLY REAPPEARS)

1. Yes
2. No
3. Refused
4. Don’t understand the question
5. Don’t know
6. **For at least the past six months, to what extent have you been limited because of a health problem in activities people usually do?**

**Would you say you have been…?**

(INTERVIEWER: READ OUT, IF NO HEALTH PROBLEM CODE AS ‘NOT LIMITED AT ALL’)

1. **Severely limited**
2. **Limited but not severely**
3. **Not limited at all**
4. Refused (DO NOT READ OUT)
5. Don’t understand the question (DO NOT READ OUT)
6. Don’t know (DO NOT READ OUT)

**THANK RESPONDENT AND TERMINATE**

1. POSTCODES BY INTERVIEWER WHERE APPLICABLE
2. District/Province (TO COMPLETE AS PER COUNTRY)
3. Gender
   1. Male
   2. Female
4. Interview Language
