# Supplementary material for: Characterising social contacts under COVID-19 control measures in Africa
Source: BMC Med. 2022 Oct 12;20:344. doi: 10.1186/s12916-022-02543-6 (PMC9553295; doi:10.1186/s12916-022-02543-6)
Supplement: Supplementary file 2 — Additional file 2: Table S1. Survey 2 Respondent Descriptives. Table S2. Population age structure. Table S3. Percentage female and rural population. Table S4. Mean, median, standard deviation, interquartile range (IQR) contacts in both surveys. Table S5. Permutation test results by main respondent characteristic and survey wave. Table S6. Oxford Stringency Index for surveys 1 and 2. Table S7. Percentage of people who agreed to participate out of total people called. [file 12916_2022_2543_MOESM2_ESM.docx]

**Table S1: Survey 2 Respondent Descriptives**

|  | Cameroon  n=1,323 | DRC  n=1,316 | Egypt  n=1,409 | Ethiopia  n=1,483 | Ghana  n=1,298 | Guinea  n=1,302 | Ivory Coast  N=1,397 | Kenya  n=1,461 | Liberia  n=1,313 | Morocco  n=1,238 | Mozambique  n=1,333 | Nigeria  n=1,318 | Senegal  n=1,353 | South Africa  n=1,639 | Sudan  n=1,380 | Tunisia  n=1,219 | Uganda  n=1,246 | Zambia  n=1,328 | Zimbab-we  n=1,284 |
| --- | --- | --- | --- | --- | --- | --- | --- | --- | --- | --- | --- | --- | --- | --- | --- | --- | --- | --- | --- |
| **Gender n (col %)** | | | | | | | | | | | | | | | | | | |  |
| Female | 646 (49%) | 648 (49%) | 680 (48%) | 721 (49%) | 640 (49%) | 633 (49%) | 649 (46%) | 653 (45%) | 530 (40%) | 582 (47%) | 694 (52%) | 639 (48%) | 625 (46%) | 868 (53%) | 723 (52%) | 607 (50%) | 635 (51%) | 657 (49%) | 663 (52%) |
| Male | 677 (51%) | 668 (51%) | 729 (52%) | 762 (51%) | 658 (51%) | 669 (51%) | 748 (54%) | 808 (55%) | 783 (60%) | 656 (53%) | 639 (48%) | 679 (52%) | 728 (54%) | 771 (47%) | 657 (48%) | 612 (50%) | 611 (49%) | 671 (51%) | 621 (48%) |
| **Age group n (col %)** | | | | | | | | | | | | | | | | | | |  |
| 19-29 | 717 (54%) | 601 (46%) | 532 (38%) | 878 (59%) | 769 (59%) | 828 (64%) | 656 (47%) | 532 (36%) | 551 (42%) | 269 (22%) | 751 (56%) | 581 (44%) | 536 (40%) | 513 (31%) | 914 (66%) | 255 (21%) | 451 (36%) | 764 (58%) | 476 (37%) |
| 30-39 | 430 (33%) | 459 (35%) | 425 (30%) | 374 (25%) | 408 (31%) | 292 (22%) | 500 (36%) | 514 (35%) | 417 (32%) | 271 (22%) | 346 (26%) | 501 (38%) | 419 (31%) | 526 (32%) | 292 (21%) | 287 (24%) | 388 (31%) | 340 (26%) | 416 (32%) |
| 40-49 | 126 (10%) | 189 (14%) | 228 (16%) | 145 (10%) | 90 (7%) | 96 (7%) | 173 (12%) | 245 (17%) | 230 (18%) | 327 (26%) | 156 (12%) | 168 (13%) | 254 (19%) | 371 (23%) | 118 (9%) | 252 (21%) | 206 (17%) | 134 (10%) | 256 (20%) |
| 50-59 | 36 (3%) | 52 (4%) | 192 (14%) | 60 (4%) | 27 (2%) | 52 (4%) | 58 (4%) | 110 (8%) | 86 (7%) | 225 (18%) | 60 (5%) | 50 (4%) | 93 (7%) | 162 (10%) | 49 (4%) | 236 (19%) | 124 (10%) | 62 (5%) | 96 (7%) |
| 60 | 14 (1%) | 15 (1%) | 32 (2%) | 26 (2%) | 4 (0%) | 34 (3%) | 10 (1%) | 60 (4%) | 29 (2%) | 146 (12%) | 20 (2%) | 18 (1%) | 51 (4%) | 67 (4%) | 7 (1%) | 189 (16%) | 77 (6%) | 28 (2%) | 40 (3%) |
| **Area** | | | | | | | | | | | | | | | | | | |  |
| Rural | 634 (48%) | 632 (48%) | 783 (56%) | 1087 (73%) | 638 (49%) | 756 (58%) | 734 (53%) | 919 (63%) | 567 (43%) | 496 (40%) | 852 (64%) | 718 (54%) | 739 (55%) | 864 (53%) | 822 (60%) | 401 (33%) | 989 (79%) | 782 (59%) | 837 (65%) |
| Urban | 689 (52%) | 684 (52%) | 626 (44%) | 396 (27%) | 660 (51%) | 546 (42%) | 663 (47%) | 542 (37%) | 746 (57%) | 742 (60%) | 481 (36%) | 600 (46%) | 614 (45%) | 775 (47%) | 558 (40%) | 818 (67%) | 257 (21%) | 546 (41%) | 447 (35%) |
| **Household head education n (col %)** | | | | | | | | | | | | | | | | | | |  |
| No education | 42 (3%) | 14 (1%) | 183 (13%) | 281 (19%) | 78 (6%) | 455 (35%) | 307 (22%) | 107 (7%) | 141 (11%) | 520 (42%) | 149 (11%) | 44 (3%) | 485 (36%) | 33 (2%) | 156 (11%) | 253 (21%) | 264 (21%) | 61 (5%) | 21 (2%) |
| Primary | 284 (21%) | 58 (4%) | 189 (13%) | 307 (21%) | 226 (17%) | 202 (16%) | 410 (29%) | 295 (20%) | 193 (15%) | 392 (32%) | 379 (28%) | 75 (6%) | 419 (31%) | 238 (15%) | 342 (25%) | 524 (43%) | 536 (43%) | 197 (15%) | 143 (11%) |
| Secondary | 295 (22%) | 251 (19%) | 396 (28%) | 179 (12%) | 525 (40%) | 61 (5%) | 197 (14%) | 460 (31%) | 365 (28%) | 118 (10%) | 458 (34%) | 400 (30%) | 192 (14%) | 774 (47%) | 159 (12%) | 138 (11%) | 175 (14%) | 297 (22%) | 495 (39%) |
| Tertiary | 279 (21%) | 604 (46%) | 609 (43%) | 605 (41%) | 439 (34%) | 446 (34%) | 256 (18%) | 552 (38%) | 543 (41%) | 97 (8%) | 308 (23%) | 732 (56%) | 210 (16%) | 496 (30%) | 594 (43%) | 185 (15%) | 248 (20%) | 683 (51%) | 508 (40%) |
| Post-graduate | 323 (24%) | 381 (29%) | 24 (2%) | 86 (6%) | 15 (1%) | 127 (10%) | 186 (13%) | 36 (2%) | 52 (4%) | 75 (6%) | 22 (2%) | 61 (5%) | 29 (2%) | 37 (2%) | 113 (8%) | 103 (8%) | 9 (1%) | 75 (6%) | 102 (8%) |
| Missing | 100 (7.6%) | 8 (0.6%) | 8 (0.6%) | 25 (1.7%) | 15 (1.2%) | 11 (0.8%) | 41 (2.9%) | 11 (0.8%) | 19 (1.4%) | 36 (2.9%) | 17 (1.3%) | 6 (0.5%) | 18 (1.3%) | 61 (3.7%) | 16 (1.2%) | 16 (1.3%) | 14 (1.1%) | 15 (1.1%) | 15 (1.2%) |
| **Age** | | | | | | | | | | | | | | | | | | |  |
| Median [IQR] | 28 [11] | 30 [12] | 33 [18] | 27 [12] | 28 [7.0] | 26 [11] | 30 [11] | 32 [14] | 32 [14] | 41 [20] | 28 [12] | 30 [10] | 32 [15] | 35 [16] | 26 [9.0] | 41 [23] | 33 [15] | 28 [11] | 33 [15] |
| **Household size** | | | | | | | | | | | | | | | | | | |  |
| Median [IQR] | 4.0 [3.0] | 6.0 [3.0] | 5.0 [2.0] | 5.0 [3.0] | 5.0 [4.0] | 7.0 [5.0] | 6.0 [4.0] | 4.0 [3.0] | 7.0 [5.0] | 5.0 [2.0] | 5.0 [3.0] | 5.0 [3.0] | 10 [7.0] | 5.0 [3.0] | 7.0 [4.0] | 4.0 [3.0] | 6.0 [4.0] | 5.0 [3.0] | 5.0 [2.0] |

Legend: The table shows the number (n) and percentage of total participants (col%) in a given country by gender, age group, household head education level, monthly income in United States Dollars, as well as the median and interquartile range (IQR) of the participants’ age and the reported size of the household they live in.

**Table S2: Population age structure**

| **Countries** | **Age groups** (% population) | | | | | | | | | | |
| --- | --- | --- | --- | --- | --- | --- | --- | --- | --- | --- | --- |
|  | **0-19** | **20-29** | | **30-39** | | **40-49** | | **50-59** | | **60+** | |
|  | **Total** | **Total** | **Adult** | **Total** | **Adult** | **Total** | **Adult** | **Total** | **Adult** | **Total** | **Adult** |
| **Cameroon** | 53% | 17% | 36% | 13% | 27% | 8% | 17% | 5% | 10% | 4% | 9% |
| **DRC** | 56% | 16% | 36% | 11% | 25% | 7% | 17% | 5% | 11% | 5% | 11% |
| **Egypt** | 42% | 16% | 28% | 15% | 26% | 11% | 19% | 8% | 13% | 8% | 14% |
| **Ethiopia** | 51% | 19% | 38% | 12% | 25% | 8% | 16% | 5% | 10% | 5% | 11% |
| **Ghana** | 47% | 17% | 33% | 13% | 26% | 10% | 18% | 7% | 13% | 5% | 10% |
| **Guinea** | 55% | 18% | 40% | 11% | 25% | 7% | 15% | 5% | 10% | 5% | 10% |
| **Ivory Coast** | 53% | 18% | 37% | 12% | 25% | 8% | 17% | 5% | 11% | 5% | 10% |
| **Kenya** | 50% | 18% | 36% | 14% | 28% | 9% | 18% | 5% | 10% | 4% | 8% |
| **Liberia** | 51% | 17% | 34% | 12% | 26% | 9% | 18% | 6% | 12% | 5% | 11% |
| **Morocco** | 35% | 16% | 24% | 15% | 23% | 12% | 19% | 10% | 16% | 12% | 18% |
| **Mozambique** | 55% | 17% | 38% | 11% | 26% | 7% | 16% | 4% | 10% | 4% | 10% |
| **Nigeria** | 54% | 16% | 35% | 12% | 26% | 8% | 18% | 5% | 11% | 5% | 10% |
| **Senegal** | 53% | 17% | 36% | 12% | 26% | 8% | 17% | 5% | 11% | 5% | 10% |
| **South Africa** | 37% | 17% | 27% | 17% | 27% | 12% | 19% | 8% | 13% | 9% | 14% |
| **Sudan** | 51% | 17% | 35% | 12% | 24% | 8% | 17% | 6% | 12% | 6% | 12% |
| **Tunisia** | 31% | 15% | 21% | 16% | 23% | 13% | 19% | 12% | 17% | 13% | 19% |
| **Uganda** | 58% | 17% | 41% | 11% | 26% | 7% | 16% | 4% | 10% | 3% | 8% |
| **Zambia** | 56% | 17% | 39% | 12% | 27% | 8% | 17% | 4% | 9% | 3% | 8% |
| **Zimbabwe** | 53% | 17% | 35% | 13% | 27% | 8% | 18% | 5% | 10% | 5% | 10% |

**Source:** United Nations. Department of Economic and Social Affairs. Population Dynamics. 2021. https://population.un.org/wpp/DataQuery/. Accessed 2 Aug 2021.

**Table S3: Percentage female and rural population**

| **Countries** | **Female population**  (% total population, 2020) | **Rural population**  (% total population, 2020) |
| --- | --- | --- |
| **Cameroon** | 50% | 42% |
| **DRC** | 50% | 54% |
| **Egypt** | 50% | 57% |
| **Ethiopia** | 50% | 78% |
| **Ghana** | 49% | 43% |
| **Guinea** | 52% | 63% |
| **Ivory Coast** | 50% | 48% |
| **Kenya** | 51% | 72% |
| **Liberia** | 50% | 48% |
| **Morocco** | 50% | 36% |
| **Mozambique** | 51% | 63% |
| **Nigeria** | 49% | 48% |
| **Senegal** | 51% | 52% |
| **South Africa** | 51% | 33% |
| **Sudan** | 50% | 65% |
| **Tunisia** | 50% | 30% |
| **Uganda** | 51% | 75% |
| **Zambia** | 51% | 55% |
| **Zimbabwe** | 52% | 68% |

**Source:**

World Bank staff estimates based on age/sex distributions of United Nations Population Division's World Population Prospects: 2019 Revision.

World Bank staff estimates based on the United Nations Population Division's World Urbanization Prospects: 2018 Revision.

**Table S4: Mean, median, standard deviation, interquartile range (IQR) contacts in both surveys**

| **Country** | **Survey 1** | | **Survey 2** | | **t-test significance on the mean** | **Permutation test** | |
| --- | --- | --- | --- | --- | --- | --- | --- |
|  | **Median (IQR)** | **Mean (SD)** | **Median (IQR)** | **Mean (SD)** |  | **Median** | **Mean** |
| **Cameroon** | 20 (41) | 41 (± 56) | 9 (14) | 16 (± 20) | **** | **** | **** |
| **DRC** | 15 (25) | 28 (± 37) | 17 (19) | 23 (± 22) | ** | * | **** |
| **Egypt** | 13 (22) | 22 (± 29) | 12 (17) | 19 (± 23) |  |  | ** |
| **Ethiopia** | 4 (8) | 9 (± 16) | 12 (20) | 21 (± 25) | **** | **** | **** |
| **Ghana** | 12 (23) | 24 (± 34) | 22 (27) | 34 (± 38) | **** | **** | **** |
| **Guinea** | 12 (30) | 26 (± 39) | 14 (23) | 25 (± 34) |  |  |  |
| **Ivory Coast** | 14 (21) | 22 (± 26) | 17 (22) | 25 (± 26) | * | *** | ** |
| **Kenya** | 13 (32) | 29 (± 39) | 14 (26) | 25 (± 30) |  |  | * |
| **Liberia** | 16 (24) | 27 (± 32) | 18 (36) | 35 (± 45) | **** |  | **** |
| **Morocco** |  |  | 8 (11) | 13 (± 15) |  |  |  |
| **Mozambique** | 11 (17) | 20 (± 30) | 12 (15) | 19 (± 22) |  |  |  |
| **Nigeria** | 12 (19) | 19 (± 24) | 15 (27) | 28 (± 38) | **** | *** | **** |
| **Senegal** | 15 (17) | 21 (± 20) | 15 (17) | 19 (± 19) |  |  | * |
| **South Africa** | 10 (16) | 17 (± 24) | 9 (14) | 16 (± 20) |  |  |  |
| **Sudan** | 24 (38) | 40 (± 47) | 33 (40) | 50 (± 53) | **** | **** | **** |
| **Tunisia** | 10 (24) | 25 (± 41) | 7 (14) | 16 (± 26) | **** | **** | **** |
| **Uganda** | 9 (14) | 16 (± 20) | 22 (32) | 34 (± 39) | **** | **** | **** |
| **Zambia** | 13 (27) | 28 (± 37) | 14 (26) | 27 (± 34) |  |  |  |
| **Zimbabwe** | 6 (11) | 14 (± 27) | 7 (10) | 13 (± 20) |  |  |  |

Legend: IQR – inter-quartile range; significance levels: **** 0.0001, *** 0.001, ** 0.01, * 0.1.

**Table S5: Permutation test results by main respondent characteristic and survey wave**

| **Country** | **Gender (male vs female)** | | | | **Location (urban vs rural)** | | | |
| --- | --- | --- | --- | --- | --- | --- | --- | --- |
|  | **Survey 1** | | **Survey 2** | | **Survey 1** | | **Survey 2** | |
|  | **Mean** | **Median** | **Mean** | **Median** | **Mean** | **Median** | **Mean** | **Median** |
| **Cameroon** | **** | **** | **** | **** | **** | * | **** | * |
| **DRC** | * | * | * |  |  |  |  |  |
| **Egypt** | **** | **** | **** | **** |  |  |  |  |
| **Ethiopia** | **** |  | *** | * |  |  | * |  |
| **Ghana** | **** | * |  |  |  |  |  |  |
| **Guinea** | *** | * | **** | **** |  |  | * |  |
| **Ivory Coast** | **** | **** |  | * | * |  |  | * |
| **Kenya** | **** | **** | **** | **** | ** | * | ** | ** |
| **Liberia** | * | * | ** | * |  |  |  |  |
| **Morocco** | - | - | **** | **** | - | - | * | * |
| **Mozambique** | **** | **** | **** | **** |  |  |  | * |
| **Nigeria** | **** |  | **** | *** | * | ** | * |  |
| **Senegal** | * | * | **** | * | **** | **** | **** | **** |
| **South Africa** |  |  | * |  |  | * |  |  |
| **Sudan** | **** | **** | **** | **** |  |  |  |  |
| **Tunisia** | **** | **** | **** | **** |  |  |  | * |
| **Uganda** | **** | * | **** | **** |  |  |  |  |
| **Zambia** | ** | * | **** | **** |  |  | *** | *** |
| **Zimbabwe** | *** | * | **** | **** |  | * |  |  |

Legend: Significance levels: **** 0.0001, *** 0.001, ** 0.01, * 0.1.

**Table S6: Oxford Stringency Index for Surveys 1 and 2**

|  | **Survey 1** | **Survey 2** |
| --- | --- | --- |
| **Cameroon** | 60.19 | 32.72 |
| **DRC** | 52.65 | 42.97 |
| **Egypt** | 62.96 | 54.63 |
| **Ethiopia** | 80.56 | 39.20 |
| **Ghana** | 52.78 | 40.12 |
| **Guinea Conakry** | 70.83 | 50.26 |
| **Ivory Coast** | 31.94 | 22.22 |
| **Kenya** | 72.16 | 50.93 |
| **Liberia** | 64.81 | 41.29 |
| **Morocco** | *-* | 76.85 |
| **Mozambique** | 70.04 | 61.73 |
| **Nigeria** | 68.38 | 58.33 |
| **Senegal** | 33.20 | 49.36 |
| **South Africa** | 80.56 | 56.48 |
| **Sudan** | 70.01 | 23.05 |
| **Tunisia** | 24.67 | 79.63 |
| **Uganda** | 79.50 | 48.66 |
| **Zambia** | 50.93 | 36.11 |
| **Zimbabwe** | 80.56 | 86.04 |

**Table S7: Percentage of people who agreed to participate out of total people called**

| **Country** | **Survey 1** | **Survey 2** |
| --- | --- | --- |
| **Cameroon** | 57% | 64% |
| **DRC** | 34% | 52% |
| **Egypt** | 15% | 49% |
| **Ethiopia** | 36% | 64% |
| **Ghana** | 65% | 81% |
| **Guinea** | 27% | 21% |
| **Ivory Coast** | 61% | 68% |
| **Kenya** | 58% | 30% |
| **Liberia** | 76% | 73% |
| **Morocco** | - | 11% |
| **Mozambique** | 20% | 20% |
| **Nigeria** | 32% | 63% |
| **Senegal** | 57% | 55% |
| **South Africa** | 28% | 39% |
| **Sudan** | 36% | 83% |
| **Tunisia** | 33% | 37% |
| **Uganda** | 31% | 36% |
| **Zambia** | 36% | 74% |
| **Zimbabwe** | 36% | 21% |
| **Average** | 41% | 50% |
