# Supplementary figures and images for: Characterising social contacts under COVID-19 control measures in Africa
Source: BMC Med. 2022 Oct 12;20:344. doi: 10.1186/s12916-022-02543-6 (PMC9553295; doi:10.1186/s12916-022-02543-6)

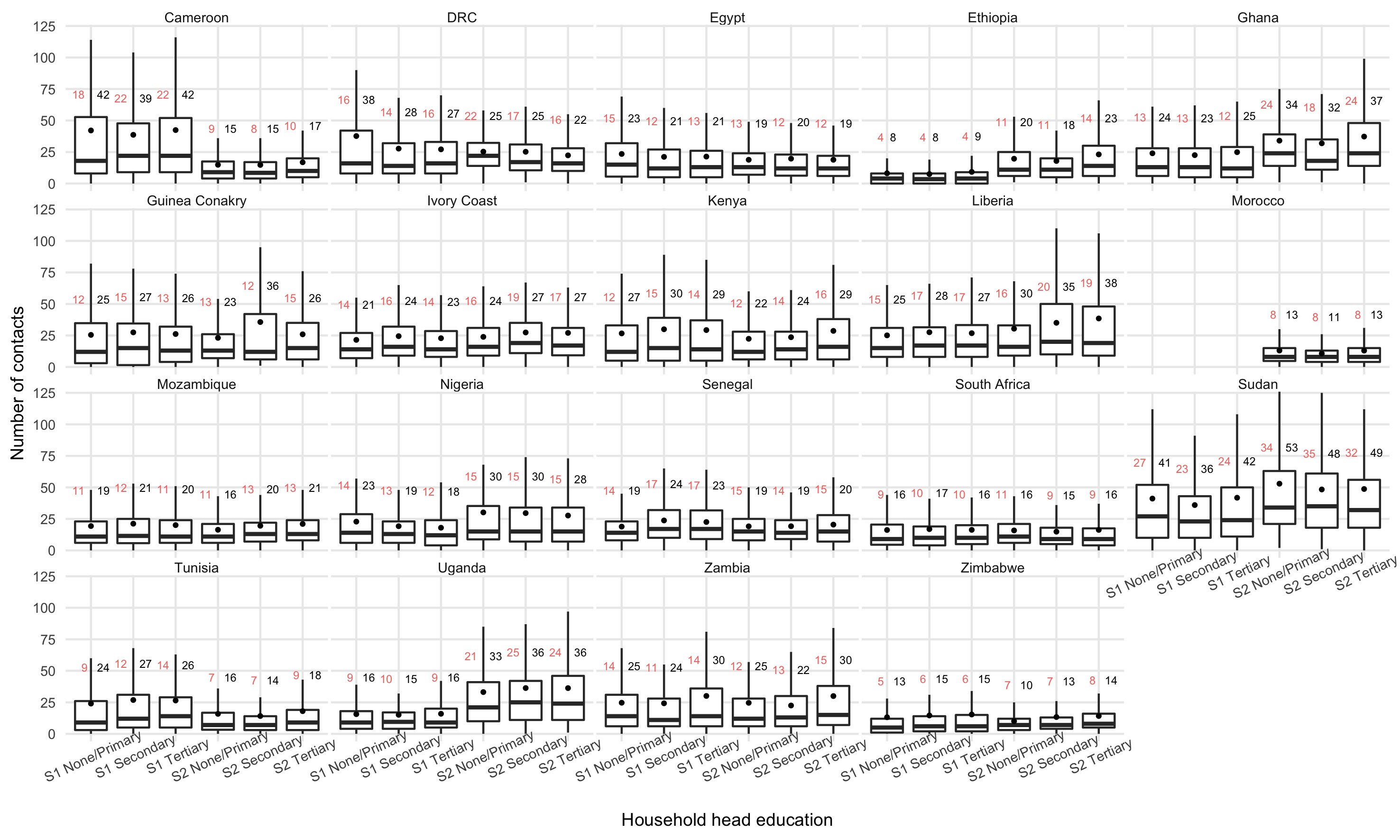

Supplement: Supplementary file 3 — Additional file 3: Fig. S1. Contacts by household head education level. [file 12916_2022_2543_MOESM3_ESM.jpg]

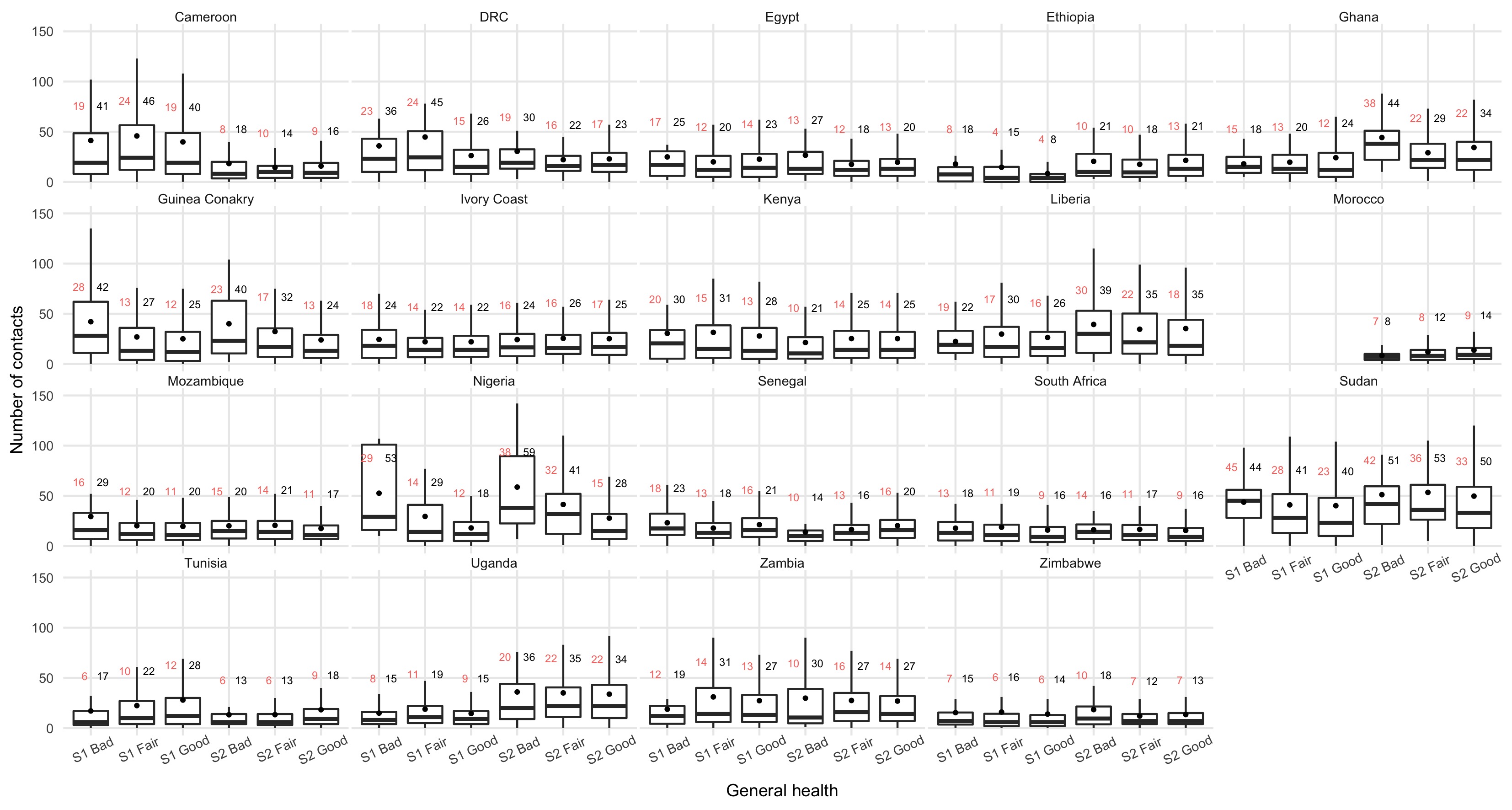

Supplement: Supplementary file 4 — Additional file 4: Fig. S2. Contacts by self-reported general health. [file 12916_2022_2543_MOESM4_ESM.jpg]

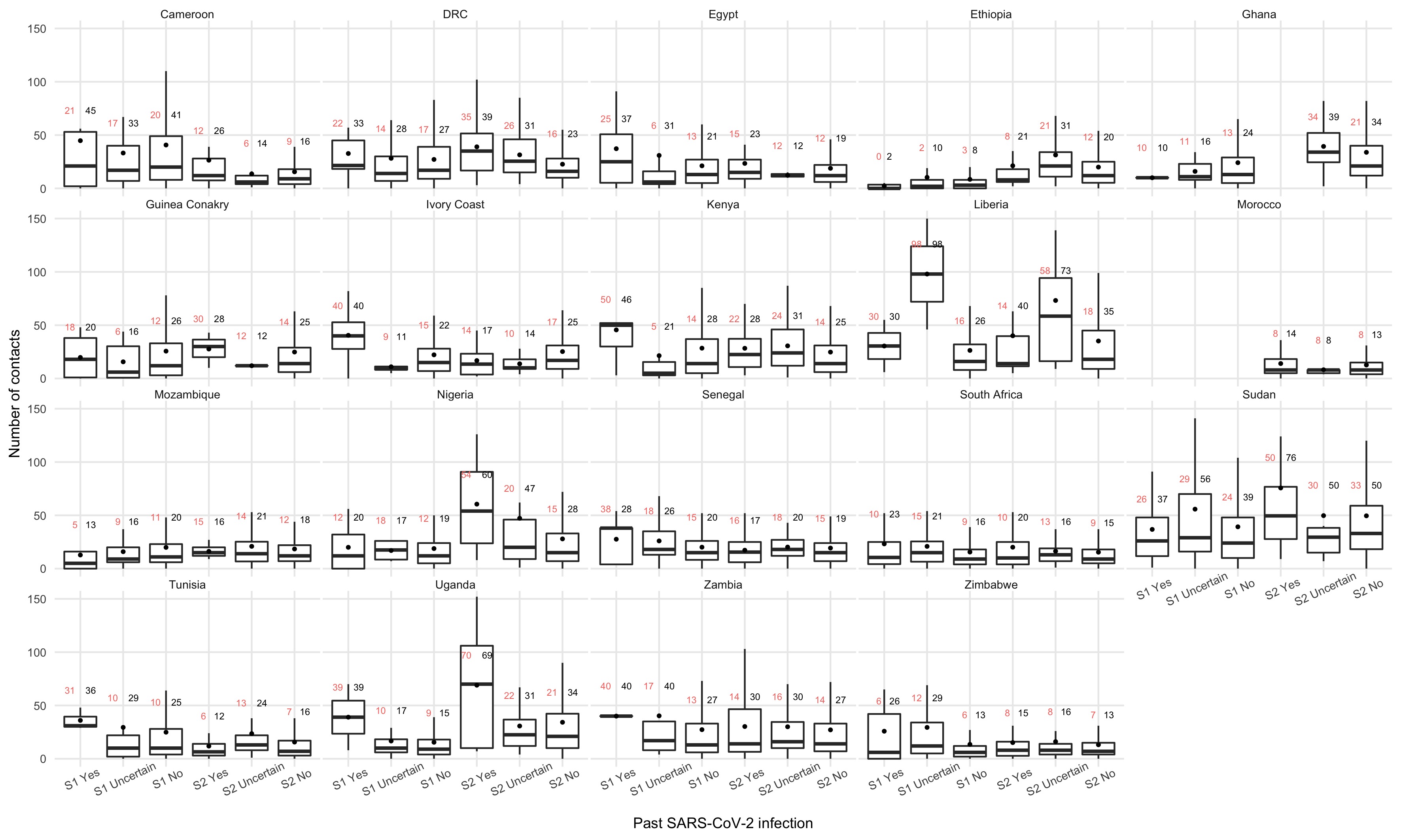

Supplement: Supplementary file 5 — Additional file 5: Fig. S3. Contacts by past SARS-CoV-2 infection status. [file 12916_2022_2543_MOESM5_ESM.jpg]

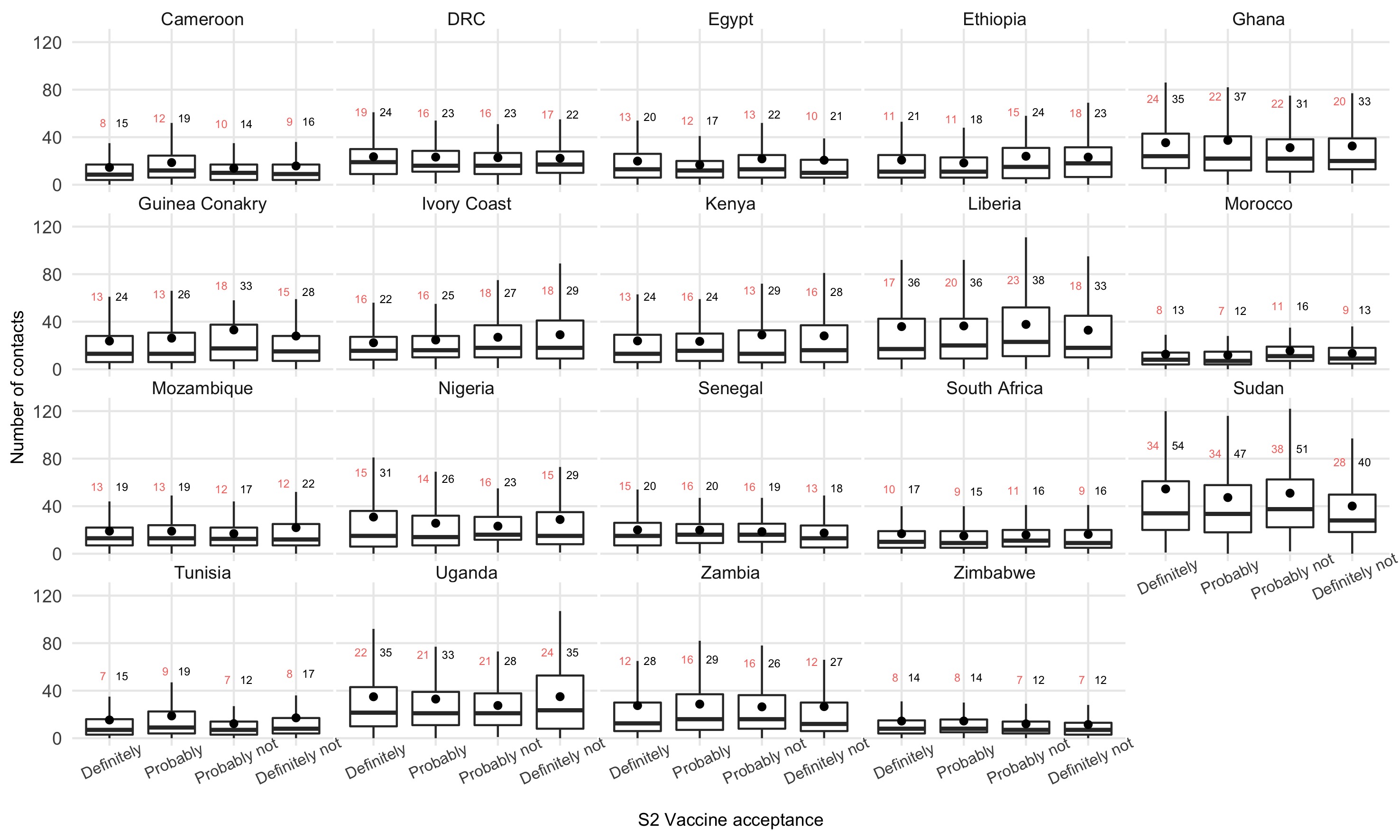

Supplement: Supplementary file 6 — Additional file 6: Fig. S4. Contacts by vaccine acceptance attitude (Survey 2 only). [file 12916_2022_2543_MOESM6_ESM.jpg]

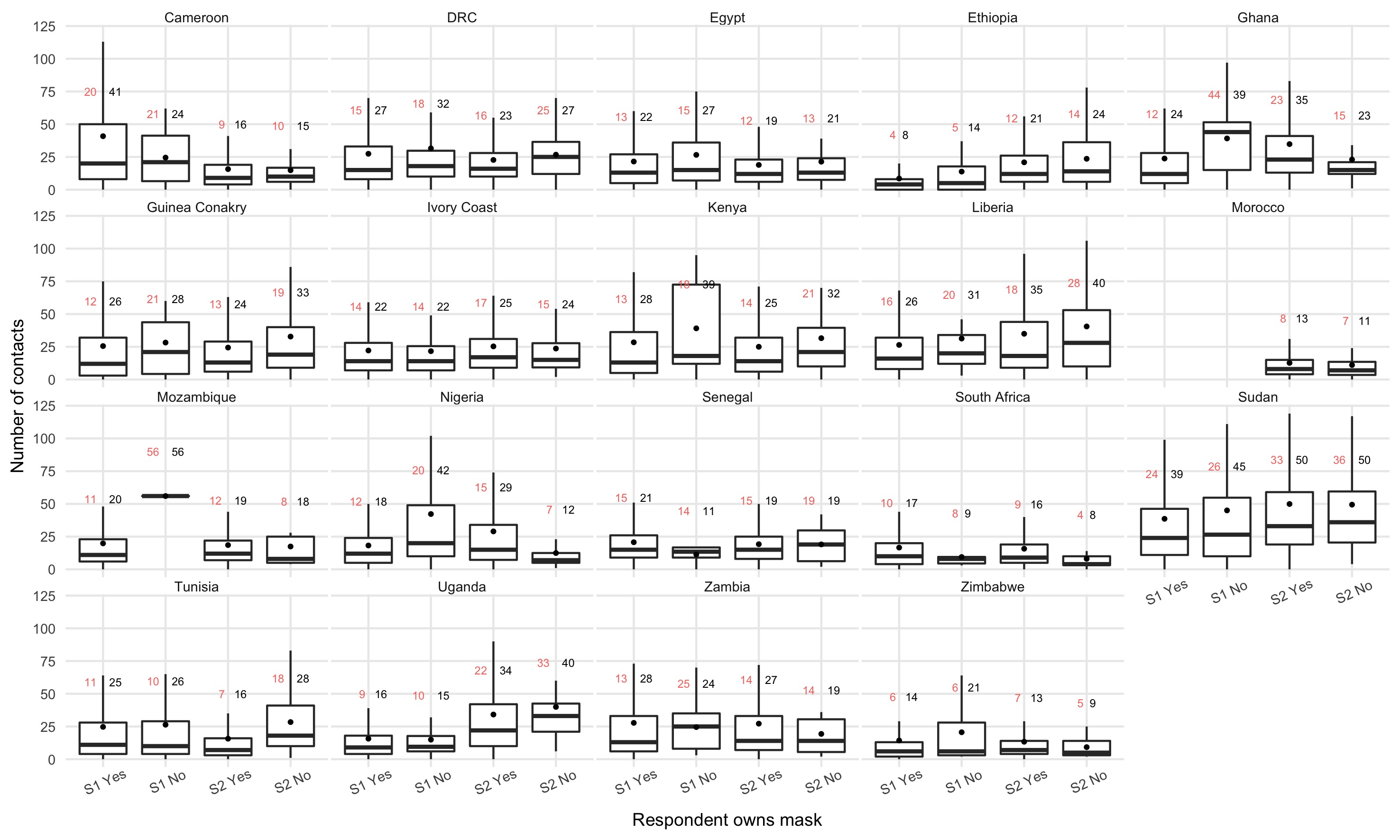

Supplement: Supplementary file 7 — Additional file 7: Fig. S5. Contacts by mask ownership. [file 12916_2022_2543_MOESM7_ESM.jpg]

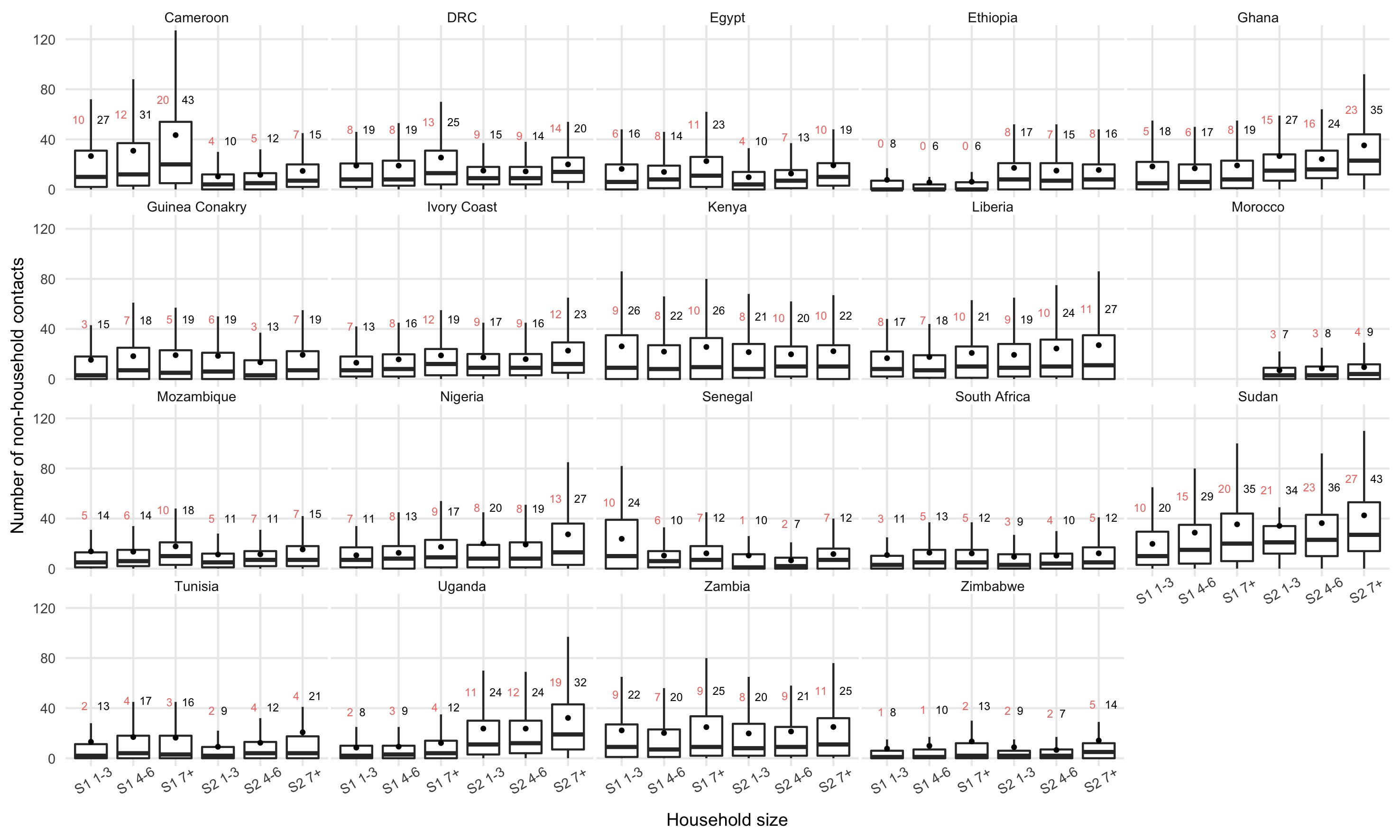

Supplement: Supplementary file 8 — Additional file 8: Fig. S6. Non-household contacts by household size. [file 12916_2022_2543_MOESM8_ESM.jpg]

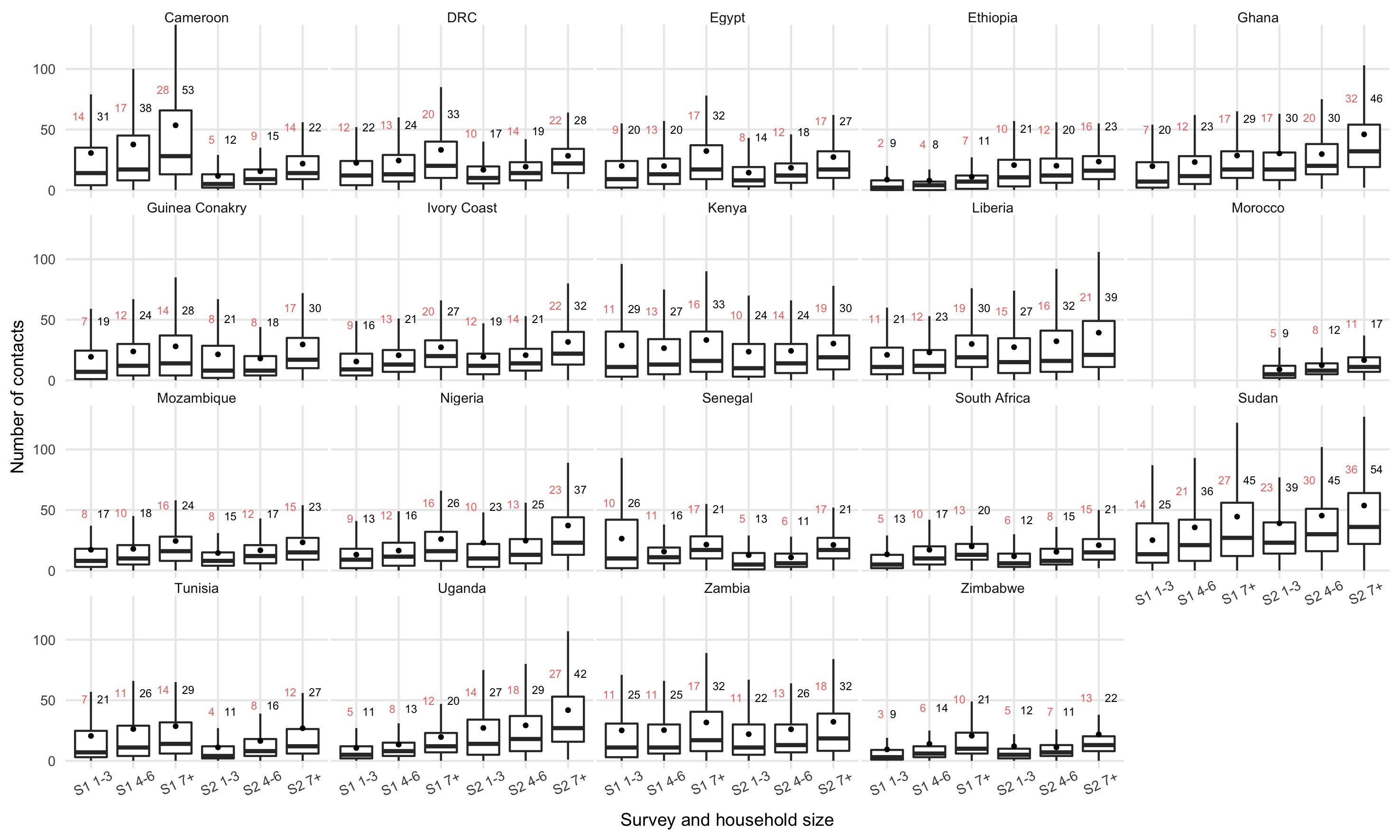

Supplement: Supplementary file 9 — Additional file 9: Fig. S7. Contacts by household size. [file 12916_2022_2543_MOESM9_ESM.jpg]

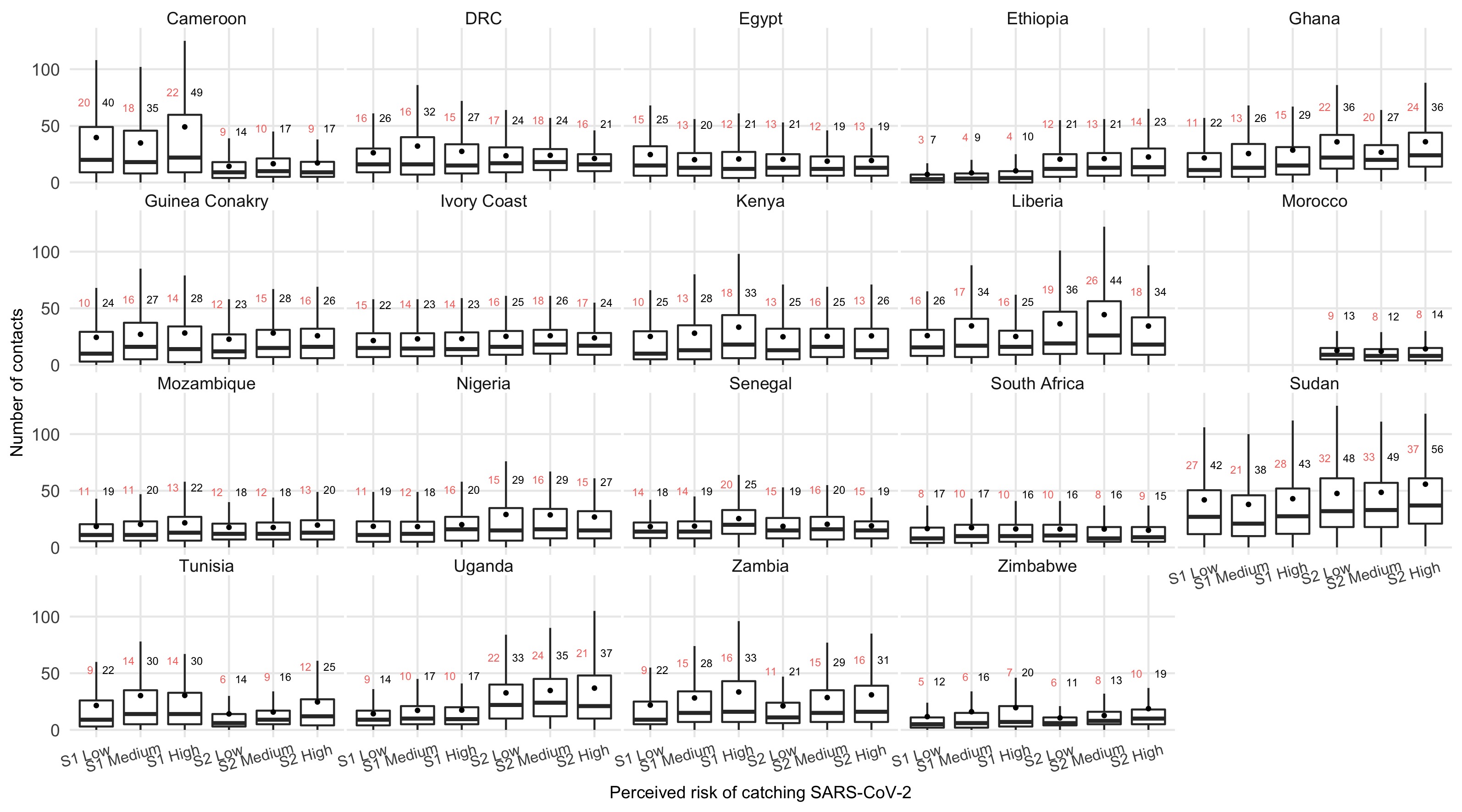

Supplement: Supplementary file 10 — Additional file 10: Fig. S8. Contacts by perceived risk of catching SARS-CoV-2. [file 12916_2022_2543_MOESM10_ESM.jpg]

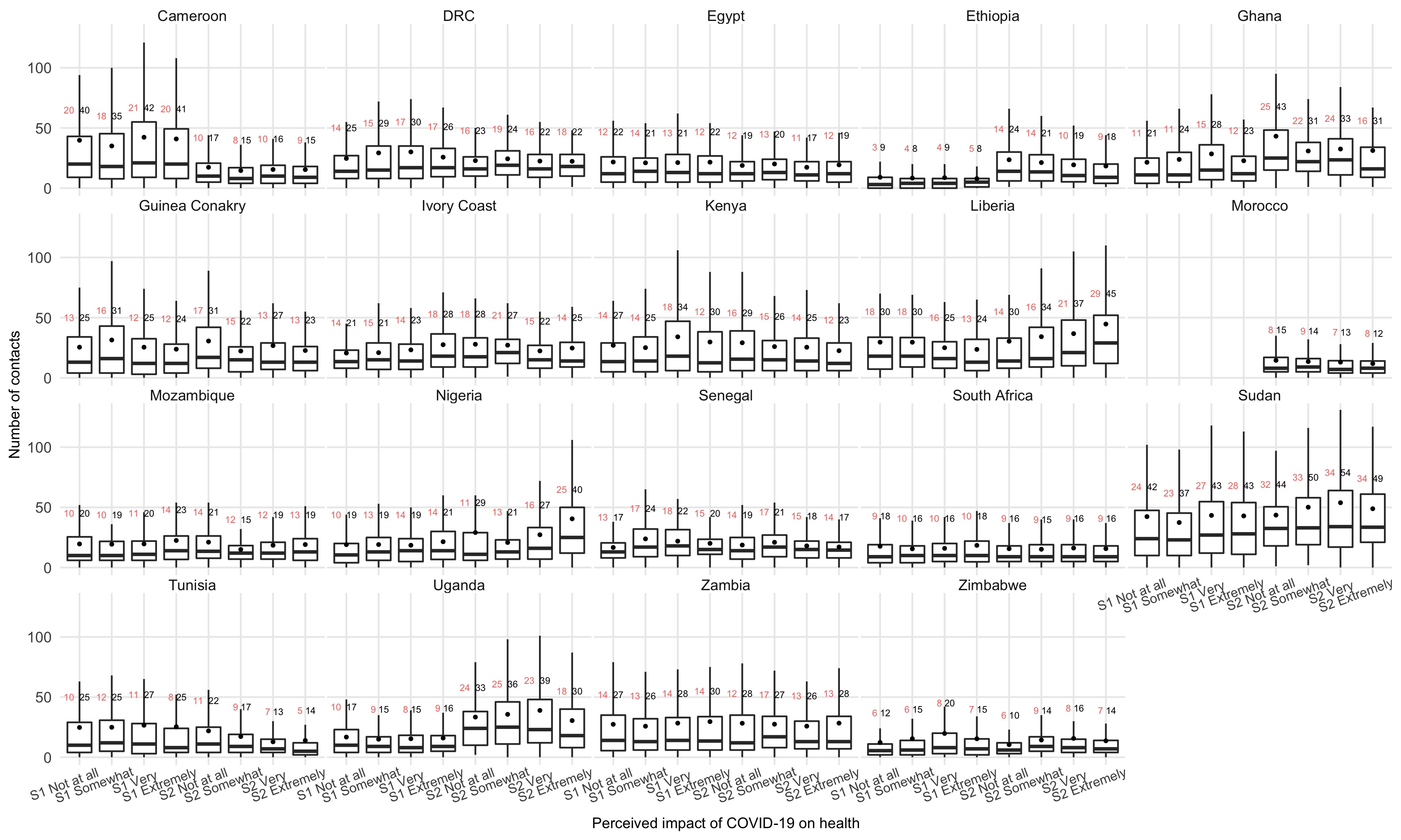

Supplement: Supplementary file 11 — Additional file 11: Fig. S9. Contacts by perceived impact of COVID-19 on health. [file 12916_2022_2543_MOESM11_ESM.jpg]

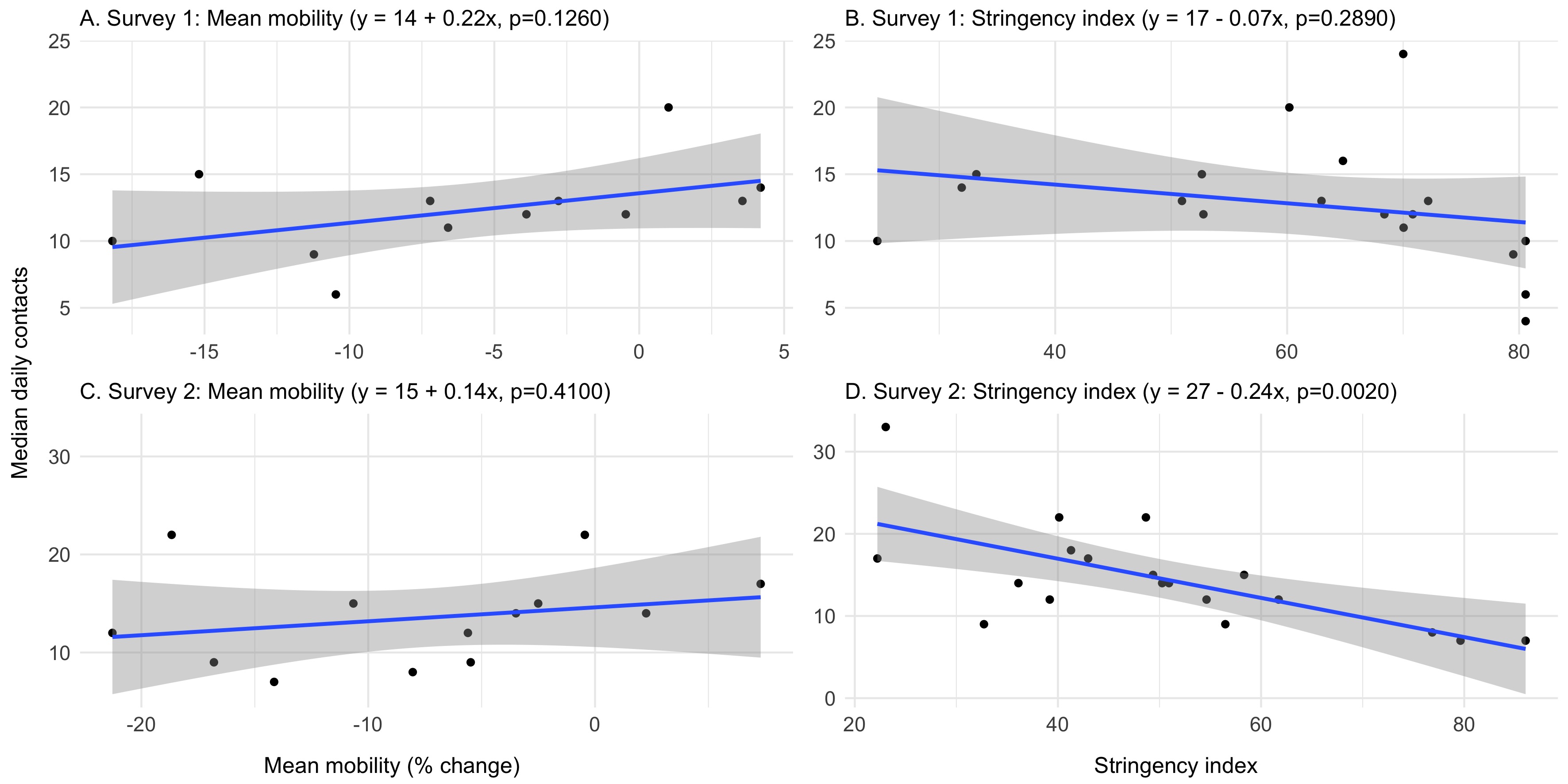

Supplement: Supplementary file 12 — Additional file 12: Fig. S10. Relationship between median contacts and change in mobility and restrictions stringency. [file 12916_2022_2543_MOESM12_ESM.jpg]
